# Enlightenment beats prejudice: The reversibility of stereotype-induced memory distortion

**DOI:** 10.3758/s13423-018-1541-7

**Published:** 2019-01-02

**Authors:** Hartmut Blank, Lauren Rutter, Rebecca Armstrong

**Affiliations:** Department of Psychology, University of Portsmouth, King Henry Building, King Henry I Street, Portsmouth, PO1 2DY UK

**Keywords:** Person memory, Social stereotypes, Warning, Reversibility

## Abstract

Social stereotypes impact how we remember people, but how stable is this influence? Inspired by the reversibility of the eyewitness misinformation effect through postwarnings about the planting of misinformation (‘enlightenment’), we explored if stereotype influence on person memory can be similarly reversed. Participants read person self-descriptions and subsequently answered memory test questions either with or without stereotype labels, establishing sizeable stereotype-induced memory distortion. One week later, the participants answered the same questions again, but half were enlightened about the earlier stereotype manipulation. This eliminated the stereotype effect and restored memory for the original information, whereas memory remained distorted without enlightenment. We discuss implications for memory distortion research and for (undermining) the self-perpetuation of stereotypes in society.

**Electronic supplementary material:**

The online version of this article (10.3758/s13423-018-1541-7) contains supplementary material, which is available to authorized users.

What we remember about people can be biased along social expectations. Numerous studies have shown that social stereotypes, like other schematic expectations, can lead to distorted remembering of person-related information (Fyock & Stangor, 1994; Leichtman & Ceci, 1995; Lenton, Blair, & Hastie, 2001; Macrae, Schloerscheidt, Bodenhausen, & Milne, 2002; Rohner & Rasmussen, 2012; Sherman & Bessenoff, 1999; Stangor & McMillan, 1992). Importantly, in a societal context, such memory distortion affords a (pseudo-)validation of the stereotypes and serves to legitimise and perpetuate them (Fyock & Stangor, 1994; Macrae et al., 2002; Martin, Cunningham, Hutchison, Slessor, & Smith, 2017; Snyder & Uranowitz, 1978; van Knippenberg & Dijksterhuis, 1996).

While most studies of stereotype influence on remembering used designs where target persons were already introduced as members of certain social groups (leading to potential stereotype influence at both encoding and retrieval), there is also research investigating *retroactive* stereotype influence, mimicking situations where the group membership of a person becomes known only after getting to know the person (e.g., learning later that a person is a lesbian, or an artist; Snyder & Uranowitz, 1978; van Knippenberg & Dijksterhuis, 1996). This latter type of design parallels the classic eyewitness misinformation design (Loftus, Miller, & Burns, 1978), in which postevent misinformation is introduced to bias accounts of previously witnessed scenes.

Of core interest for the present research, memory distortion through misinformation is not inevitable, but can often be limited after the fact by *postwarning* participants about the earlier presence of misinformation. In a meta-analysis of 25 studies, Blank and Launay (2014) found that postwarnings reduced the misinformation effect to less than half of its size on average, with some warnings (e.g., the ‘enlightenment’ technique introduced by Blank, 1998; see below) being more effective than others. Moreover, it has been demonstrated that postwarnings can not only prevent participants from falling for misinformation but can also reverse an earlier misinformation effect (Oeberst & Blank, 2012). The latter research followed the typical misinformation design (i.e., Stage 1: original information, e.g., a video; Stage 2: misinformation; Stage 3: memory test) and established the presence of a misinformation effect, but then added an enlightenment-type postwarning and a further memory test. In three experiments, the initially established misinformation effect was completely reversed (in the third experiment, even after 5 weeks).

In the present research, we applied this logic to stereotype influence on memory and explored if a parallel effect reversal can be achieved by using enlightenment in a stereotype influence setting. Participants first read person information, then a stereotype was introduced just before answering a memory test. This corresponded to a standard retroactive stereotype influence design (Snyder & Uranowitz, 1978; van Knippenberg & Dijksterhuis, 1996), and we expected to find stereotypical memory distortion. After 1 week, participants returned, and half of them were told that we had made up the stereotypical labels applied to the featured persons (i.e., we revealed the earlier manipulation to these participants, like in a debriefing, but already within the experiment). Then, all participants answered the same memory test again. If enlightenment works with stereotypes, then we should expect a similar effect reversal as in Oeberst and Blank’s (2012) misinformation study.

## Method

The research included three pilot studies (see the Materials section) and a main study. The pilot studies served to (1) select highly stereotypical occupations (in the UK) for an effective stereotype influence manipulation, (2) produce stereotypical information for the person descriptions, and (3) provide stereotypicality ratings for memory test answers.

### Participants and design

Combining a student (*n* = 30) and a general population (*n* = 38) subsample yielded an initial total of 68 participants. To counterbalance conditions within each subsample, the data from four participants were not used for analysis; thus, the final sample consisted of 64 participants (39 female and 25 male; mean age = 25.6 years, range: 18–59 years), 28 of which were introductory psychology students (16 female, 12 male; mean age = 20.6 years, range: 18–30 years), and 36 were members of the general public (23 female, 13 male; mean age = 29.5 years, range: 19–59 years). Using two different subsamples was a matter of convenience, but it increases the generalisability of our findings.

We investigated stereotype influence through providing an occupation label for one of two target persons (but not for the other—counterbalanced across participants). Memory for the target person information was assessed after 20 min and again 1 week later. Before the second memory test, half of the participants were enlightened on the earlier occupational label manipulation (orthogonal to the students/general public split). These variables formed a 2 (occupational label: yes/no, within) × 2 (assessment: Time 1/Time 2, within) × 2 (enlightenment: yes/no, between) design. There were two dependent variables, stereotypical memory distortion and accuracy (see the Measures section).

### Materials

We created self-descriptions of two male characters (*Alan* and *Greg*) in a real-life context, specifically, an actual dating website (Match.com), for which two of us had registered for the purposes of the study. These profiles followed a standard preset format, a mixture of free descriptions under headings such as ‘a few words about me’ and checklist-type information (e.g., relationship status, height, weight, appearance, astrological sign; screenshots of the profiles used for the main study are available as Supplemental Material). The profiles also included a photograph each, showing *Alan* or *Greg* as moderately attractive men.

Each profile was constructed to be relevant to a particular social stereotype, based on the input from three pilot studies. In the first pilot study, we asked *N* = 10 participants to pick the two most stereotypical out of a list of 10 UK-typical occupations; these were builder (later assigned to *Greg*) and vicar (a Church of England priest, assigned to *Alan*), chosen by 60% and 80% of the judges, respectively. The second pilot study identified plausible content for *Alan*’s and *Greg*’s Match.com profiles, to be probed later in the memory tests. A further 16 participants described traits, behaviours, activities, possessions, values, likes and dislikes, as well as appearance, social class, income, relationship status, and smoking and drinking habits typically associated with the occupations of a builder and a vicar. The most frequently mentioned content was then incorporated into the dating profiles and/or the memory tests (as response alternatives), along with more neutral, stereotype-unrelated information. The profiles did not include any occupation-related information, as this was to be used later for the stereotype induction.

The memory tests for the profiles consisted of 20 questions each, probing factual information from their dating profiles (e.g., “What does Alan do every Friday night?”—the full tests are available as Supplemental Material). All questions had four substantial response alternatives, with varying degrees of stereotypicality. The latter was assessed in a third pilot study. We asked another 20 participants to “imagine there is a vicar called Alan [or a builder called Greg]; you know no other information about him apart from his occupation” and to rate the response options “purely based on how stereotypical you think they are” in relation to the respective occupation, on a scale from 0 (*least stereotypical*) to 5 (*most stereotypical*). For illustration, for the question “What does Alan do every Friday night?”, the four alternative responses and their stereotypicality ratings were “watch game shows” = 1.6, “go clubbing” = 0.2, “go to his local pub” = 1.8, and “community work” = 4.1. These ratings across all test questions were used to determine the degree of stereotypical memory distortion in the main study (see the Results section).

### Procedure

Participants consented to participate in a two-session study investigating memory for person information on websites. They were tested individually or in groups up to eight in quiet university or everyday settings. Session 1 included three phases: the dating profiles, a filler task, and memory tests for the profiles. Instructions and materials were presented via an automatically timed PowerPoint presentation (shown on PCs/laptops or a projection screen in group sessions), augmented by experimenter (L.R. or R.A.) clarifications as needed. The presentation started with an overview of the first session and then explained that participants would now see two personal profiles from a dating website (each spread across three slides) and were to study them carefully; each profile would be shown for 6 minutes (2 min per slide). After the profiles had been presented, participants worked on filler tasks (two problem-solving tasks and a sudoku puzzle) for 20 min and then completed two memory tests (one for Alan and one for Greg); they were allowed 8 min for each test.

The two memory tests were provided as separate booklets, with the order of presentation (*Alan* first, then *Greg*, or the reverse) counterbalanced. Participants were asked to circle one of four answer options for each test question and also to provide confidence ratings for their choices, on a 1 to 5 scale. On the front page of each booklet was a box containing the same photograph of the person as was shown on the dating profile, as “a reminder of who Alan [Gregory] is”, alongside other information (two lines stating, e.g., “username: Alan_d_509” and “gender: male”) in the right-hand part of the box. The sole purpose of this was to provide credibility and context for the crucial stereotype manipulation: The box of one of the booklets contained a third line stating “occupation: vicar” (or “occupation: builder”). This was always provided in the *second* booklet; our concern was that providing occupation information in the first booklet would lead participants to expect occupation information in the second booklet as well, and encourage speculations that might influence their test answers in unpredictable ways (e.g., based on imagined stereotypical occupations).

Session 2 took place exactly 7 days after Session 1. Participants filled out the memory tests once again (again, 8 min for each), but, crucially, half of them (orthogonal to the counterbalancing split) were enlightened on the earlier stereotype manipulation before answering the tests. Specifically, the experimenter explained that the occupation information about *Alan* (or *Greg*) on the front page of the memory test in the first session was incorrect and we had made it up. We then asked them to complete the memory tests, as accurately as possible, with this new information in mind. Participants in the no-enlightenment group were simply told to answer the memory tests again based on the information presented about *Alan* and *Greg* in the first session. All instructions in Session 2 were delivered orally (i.e., the wordings given here are approximate, but very close). Note that the occupational labels used in Session 1 were removed from all test booklets in Session 2 (leaving them there after enlightening participants that they were made up would have been strange, and for comparability we discarded them in the no-enlightenment group as well). Upon finishing the tests, we fully debriefed all participants and asked them not to discuss the true nature of the study with potential future participants.

## Results

### Measures

Our analysis focused on two key dependent variables, (1) stereotypical memory distortion and (2) accuracy. We determined stereotypical memory distortion in two steps. First, for each participant, we added up the stereotypicality ratings (see above) for their *chosen* answers to the 20 memory test questions for *Alan* and *Greg*. Then, to create more easily interpretable values and to control for slight differences in the stereotypicality of the response options for *Alan* and *Greg*, we divided these raw scores by the maximum possible stereotypicality score (60.6 for *Alan* and 71.1 for *Greg*, respectively, if always the most stereotypical answer was chosen). This produced *relative* stereotypicality scores, expressed as percentages of the maximum possible stereotypical memory distortion for *Alan* and *Greg*. The second dependent variable (accuracy) was the percentage of correct answers across the 20 memory test questions for *Alan* and *Greg*.

Note that our dependent variables are not redundant—a correct answer was not always low in stereotypicality. Specifically, when ranking the answer options for each of the 40 memory test questions by stereotypicality, the correct answer occupied Rank 1 (highest stereotypicality) eight times, 13 times Rank 2, 11 times Rank 3, and eight times Rank 4 (lowest stereotypicality). In this sense, stereotypical memory distortion and accuracy were separate aspects of memory performance. Still, the *effects* of our stereotype manipulation on these measures were connected. If occupational labels produce stereotypical memory distortion, then this must come at the expense of accuracy. On the other hand, any *reduction* in memory stereotypicality after enlightenment need not necessarily reinstate memory accuracy (the memory might have been permanently impaired); this is an open question.

### Stereotypical memory distortion

**Fig. 1:**
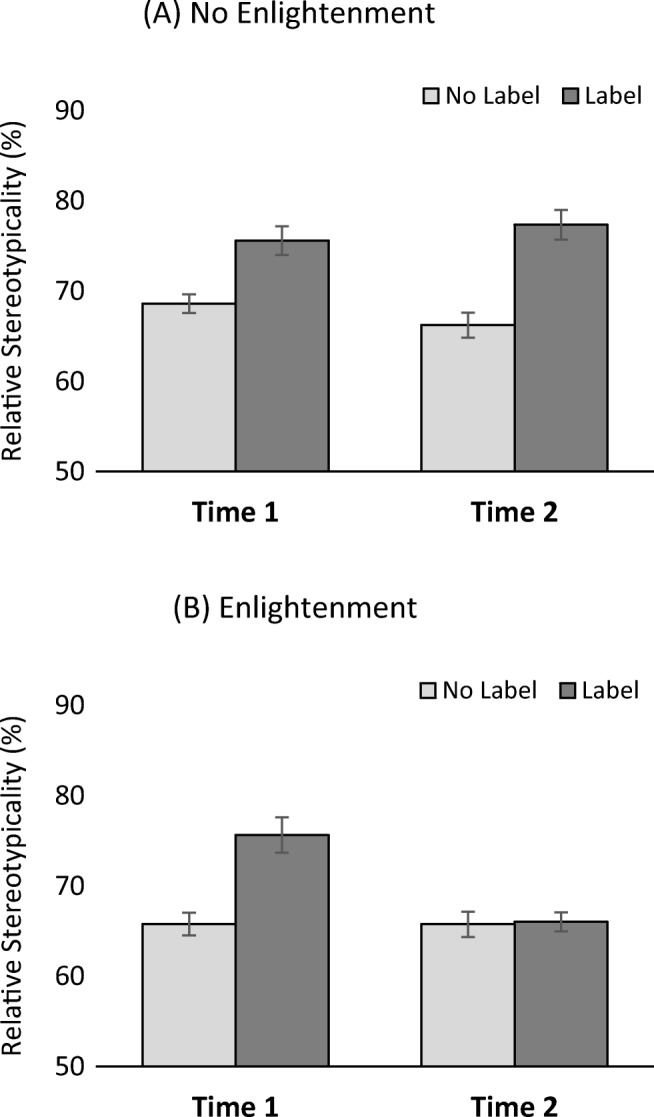
Stereotypical memory distortion as a function of enlightenment. **a** Standard effect of the stereotype/occupational label at both measurement times, **b** Stereotype effect disappears after participants are enlightened on the earlier stereotype manipulation. Error bars represent standard errors of the mean

We conducted a three-way mixed ANOVA to analyse participants’ relative stereotypicality scores as a function of occupational label (yes/no, within), assessment (Time 1/Time 2, within) and enlightenment (yes/no, between).^1^ This yielded main effects of all three variables: occupational label, *F*(1, 62) = 21.71, *p* < .001, η_p_^2^ = .26; assessment: *F*(1, 62) = 11.41, *p* = .001, η_p_^2^ = .16; enlightenment: *F*(1, 62) = 17.16, *p* < .001, η_p_^2^ = .22; an interaction between assessment and enlightenment, *F*(1, 62) = 8.83, *p* = .004, η_p_^2^ = .12, and, most important, a three-way interaction, *F*(1, 62) = 21.37, *p* < .001, η_p_^2^ = .26 (shown in Fig. 1). Further exploration of this interaction revealed that the stereotype/occupational label effect persisted (and descriptively increased; see Fig. 1) in the no-enlightenment group, Time 1, *F*(1, 31) = 10.42, *p* = .003, η_p_^2^ = .25; Time 2: *F*(1, 31) = 20.49, *p* < .001, η_p_^2^ = .40, while in the enlightenment group it was completely eliminated after the enlightenment, Time 1, *F*(1, 31) = 12.76, *p* = .001, η_p_^2^ = .29; Time 2, *F*(1, 31) = 0.02, *p* = .90, η_p_^2^ = .00.

To provide some context for the scores shown in Fig. 1, *correct* test answers (for *Alan* and *Greg*) had a relative stereotypicality score of 64.4% on average. Interestingly, this benchmark was surpassed to some degree in *all* conditions (even the no-label control conditions), which might reflect weak and unintended spreading stereotype effects due to the mere inclusion of stereotype-related material in *Alan’s* and *Greg’s* dating profiles. More important, the stereotype/occupational label conditions clearly revealed memory distortion above and beyond this level (as shown by one-sample *t* tests, *df* = 31, against the 64.4% benchmark; smallest *t* = 5.72), except of course after enlightenment (*t* = 1.51).

### Accuracy

**Fig. 2:**
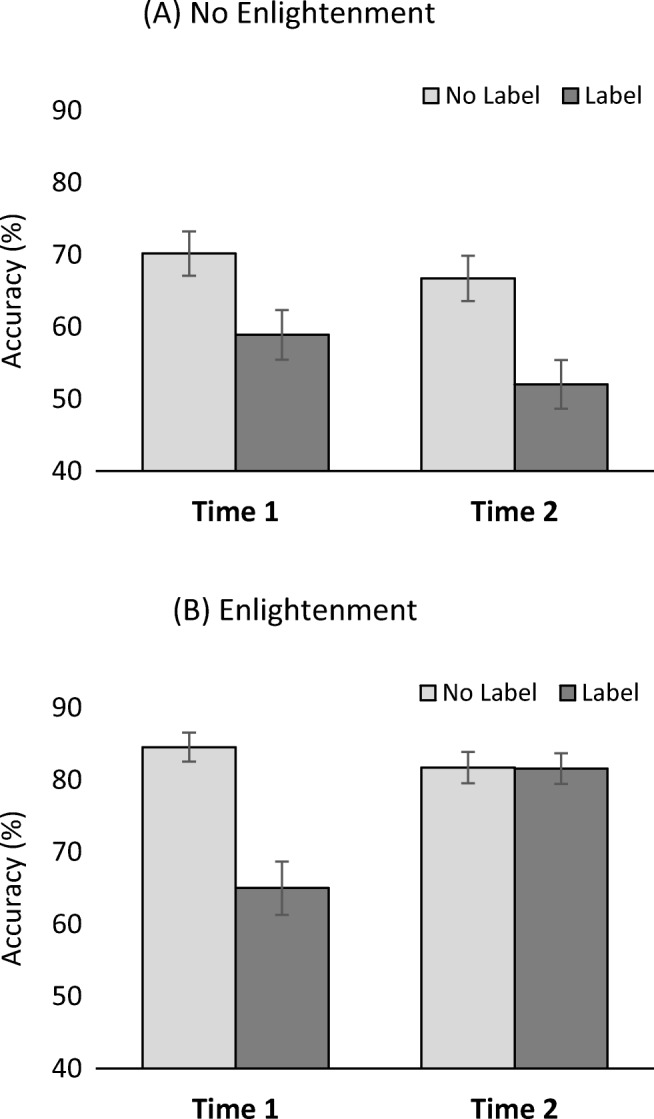
Accuracy as a function of enlightenment. **a** Standard effect of the stereotype/occupational label at both measurement times. **b** Stereotype effect disappears after participants are enlightened on the earlier stereotype manipulation. Error bars represent standard errors of the mean

A corresponding three-way ANOVA of participants’ accuracy scores revealed a complementary pattern of results. Besides main effects of occupational label, *F*(1, 62) = 38.44, *p* < .001, η_p_^2^ = .38, and enlightenment, *F*(1, 62) = 26.98, *p* < .001, η_p_^2^ = .30, as well as interactions between enlightenment and assessment, *F*(1, 62) = 15.78, *p* < .001, η_p_^2^ = .20, and enlightenment and occupational label, *F*(1, 62) = 8.64, *p* = .005, η_p_^2^ = .12, there was again a three-way interaction, *F*(1, 62) = 17.70, *p* < .001, η_p_^2^ = .22 (displayed in Fig. 2). Unpacking of the interaction showed that the stereotype/occupational label effect on accuracy persisted (and descriptively increased; see Fig. 2) in the no-enlightenment group, Time 1, *F*(1, 31) = 8.13, *p* = .008, η_p_^2^ = .21; Time 2: *F*(1, 31) = 19.73, *p* < .001, η_p_^2^ = .39, while it was—as for stereotypical memory distortion—completely eliminated in the enlightenment group, Time 1, *F*(1, 31) = 29.87, *p* < .001, η_p_^2^ = .49; Time 2, *F*(1, 31) = 0.01, *p* = .92, η_p_^2^ = .00.^2^

### Accuracy as a function of information stereotypicality

So far, we found that stereotypical memory distortion decreased and accuracy increased after enlightenment. This might mean that enlightenment restores unbiased access to the original person information. Alternatively, a *hypercorrection* mechanism (i.e., simply reversing the stereotypical bias) could produce a similar overall pattern.^3^ That is, after enlightenment, people would pick answers that are *less* stereotypical, which would boost memory performance (perhaps even beyond the level of the no-stereotype control condition) for low-stereotypical correct answers but produce an opposite effect for correct answers that are highly stereotypical. Theoretically, these effects might combine in the stereotype condition to equal control performance.

To test the contrasting explanations, we analysed accuracy after enlightenment at Time 2 as a function of the stereotypicality rank of the correct answer. In a corresponding 4 (stereotypicality rank) × 2 (occupational label) ANOVA, hypercorrection translates into a predicted interaction between these factors (more specifically, a linear trend: Accuracy should increase from most to least stereotypical, relative to the no-label control). By contrast, our interpretation that enlightenment just removes bias and restores access to original information is independent of the stereotypicality of this original information and does not predict an interaction.

**Fig. 3:**
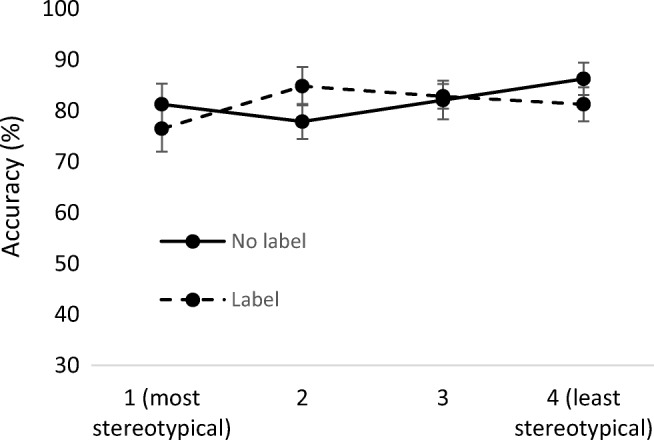
Accuracy after enlightenment as a function of (1) the stereotypicality rank of the correct answer to a memory test question and (2) the previous presence or absence of stereotype influence (occupational label vs. no label)

The data did not support the hypercorrection idea: There was little evidence for a stereotypicality Rank × Occupational Label interaction, *F*(3, 93) = 1.60, *p* = .19, η_p_^2^ = .05, let alone a linear trend, *F*(1, 31) = 0.09, *p* = .76, η_p_^2^ = .003.^4^ Instead, accuracy was more or less flat across stereotypicality rank (see Fig. 3), in line with the idea that enlightenment corrects (but not overcorrects) the stereotype influence and restores access to original person information.

## Discussion

To summarise, our participants’ memory for *Alan* and *Greg* was strongly biased in the direction of the stereotypic occupational labels provided for them at the first assessment, at the expense of accuracy. The size of both effects was large (η_p_^2^ > .20). In the no-enlightenment group, the effects persisted (and increased in size) at the second assessment 1 week later, even without a reminder of *Alan*’s or *Greg*’s occupation. In the enlightenment group, however, when participants learned that the occupations were made up and did not actually apply to *Alan* and *Greg*, the stereotypical bias disappeared, and memory accuracy was restored to the level of the control condition (in fact, both effects were reduced to η_p_^2^ = .00),^5^ and this held across all levels of stereotypicality of the original person information.

The main purpose of our study was to empirically demonstrate the reversibility of stereotype-induced memory distortion; it was not designed to isolate specific processes underlying this effect reversal. Nevertheless, a few comments are in order. Principally, the reversal of the effects would be due to the disruption of any processes that initially produced the memory distortions. Candidate mechanisms discussed in the literature include (a) stereotype-consistent response biases (in itself a heterogeneous category including, e.g., implicit associations, fluency misattribution, heuristic source monitoring, or intentional guessing; see coverage by Lenton et al., 2001; Payne, Jacoby, & Lambert, 2004; Rohner & Rasmussen, 2012; Sherman & Bessenoff, 1999; Stangor & McMillan, 1992), and (b) biased memory retrieval (i.e., using the stereotype as a cue for retrieving person information, which may facilitate access to stereotype-consistent information and/or inhibit access to stereotype-inconsistent information; e.g., Snyder & Uranowitz, 1978; van Knippenberg & Dijksterhuis, 1996). Along these lines, unbiased memory performance in our study may have been restored through disrupting (but not overcorrecting; see our accuracy-by-stereotypicality analyses) stereotype-consistent biases and/or stereotype-guided retrieval.

### Theoretical and practical implications

The present findings are generally good news both in terms of the reliability of memory and the implications for stereotype perseverance. Theoretically, the present findings, combined with Oeberst and Blank’s (2012) parallel findings on the reversibility of the eyewitness misinformation effect, suggest that previous views of the suggestibility of memory may have been too extreme. In a popular metaphor, memory has been described as malleable (Loftus, 1979), just like a piece of metal hammered on the anvil. While this is (to a considerable degree, in the light of the massive body of research supporting it) an apt metaphor, the present research invokes an alternative metaphor of memory as ‘memory metal’—a metal that can be hammered out of shape but will spring back into its original shape under suitable conditions (see, e.g., Kauffman & Mayo, 1993, for an overview). Likewise, it seems to be possible for a memory to regain—under suitable conditions such as enlightenment—its original form after having been distorted. Of course these two metaphors complement, rather than contradict, each other. But certainly, metaphors guide our views of phenomena and how we research them, and in this sense the alternative metaphor proposed here, with its emphasis on memory resilience, is a welcome antidote to the current focus on memory distortion and malleability (see, e.g., Nash & Ost, 2017).

Regarding practical consequences of the reversibility of stereotype-conserving memory distortion, the message is perhaps that attention needs to be paid to detail. Firstly, we acknowledge that the present research applies to only one kind of real-life situations—namely, when stereotypes are introduced after the fact. We cannot be sure that the present findings would extend to situations where stereotypes already influence the encoding of person information. Moreover, our enlightenment manipulation undermined the *applicability* of a stereotype to a particular case, not the stereotype itself. While there are certainly real-life equivalents of this (e.g., cases of mistaken identity or group membership), it would seem much harder to undermine stereotype influence when such a move is not possible (but still feasible in principle; e.g., Todd, Galinsky, & Bodenhausen, 2012, showed that memory distortion due to an unmistakable stereotype—race—can be limited through asking people to adopt the perspective of the targeted protagonist). In any case, the current demonstration that it is possible to completely undo stereotype influence on memory under some circumstances may encourage future research to explore the circumstances under which the preservation of stereotypes can be disrupted more generally.

#### Author note

We are grateful to Ryan Fitzgerald, Jonathan Koppel and Beatriz López for helpful comments on a draft.

## Electronic supplementary material

ESM 1 (ZIP 2.02 mb)

## Footnotes

1 We also conducted——as a check of the robustness of our findings——three-way ANOVAs on the basis of the whole initial sample of 68 participants (i.e., with imperfect counterbalancing), and using three different random realisations of a counterbalanced 64-participants sample (i.e., dropping different sets of participants from some cells to achieve counterbalancing). These analyses led to highly similar results. The version we report here is the one that was in between the two others for most of the main statistics. Further, parallel analyses to the ones we report here, but incorporating the confidence ratings from the memory tests (e.g., accuracy scores qualified by confidence), led to highly similar findings and are therefore not reported.

2 It is worth noting that both our accuracy and stereotypical memory distortion findings did not depend on whether *Alan* or *Greg* was the target of the stereotype induction (i.e., on the counterbalancing condition) or on the subsample of participants (students or general public). In respective extended ANOVAs, the critical three-way interactions shown in Figs. 1 and 2 were not compromised by higher-order interactions involving these additional variables, suggesting some generalisability of our findings.

3 We thank reviewer Bob Belli for proposing this alternative.

4 We also conducted separate *t* tests for each stereotypicality rank, comparing accuracy in the label and no-label conditions. Even without Bonferroni correction, none of these comparisons were significant (lowest *p* = .07, for Rank 2).

5 In the context of the current replication debate (e.g., Zwaan, Etz, Lucas & Donnellan, 2018), it is also worth mentioning that the present findings broadly replicate——and partly bring out more convincingly——a similar pattern of findings obtained 15 years earlier in a different culture (Germany), but with a largely identical design (Blank, 2001, Chap. 6). The findings paralleled the present study for stereotypical memory distortion, but, for accuracy, the critical interaction was weaker (*p* = .13). This was likely owed to a smaller effect size, resulting from different and less reliable measurement (only a few items measured accuracy). In this sense, the present study (using more reliable accuracy measurement) is a higher-powered replication of Blank (2001).
