# Supplementary material for: Enlightenment beats prejudice: The reversibility of stereotype-induced memory distortion
Source: Psychon Bull Rev. 2019 Jan 2;26(3):1001–7. doi: 10.3758/s13423-018-1541-7 (PMC6557864; doi:10.3758/s13423-018-1541-7)
Supplement: Supplementary file 1 — (ZIP 2.02 mb) [file 13423_2018_1541_MOESM1_ESM.zip › supplementary 11-27-18/Instructions and materials - order 2 (vicar then builder).pptx]

## Slide 1
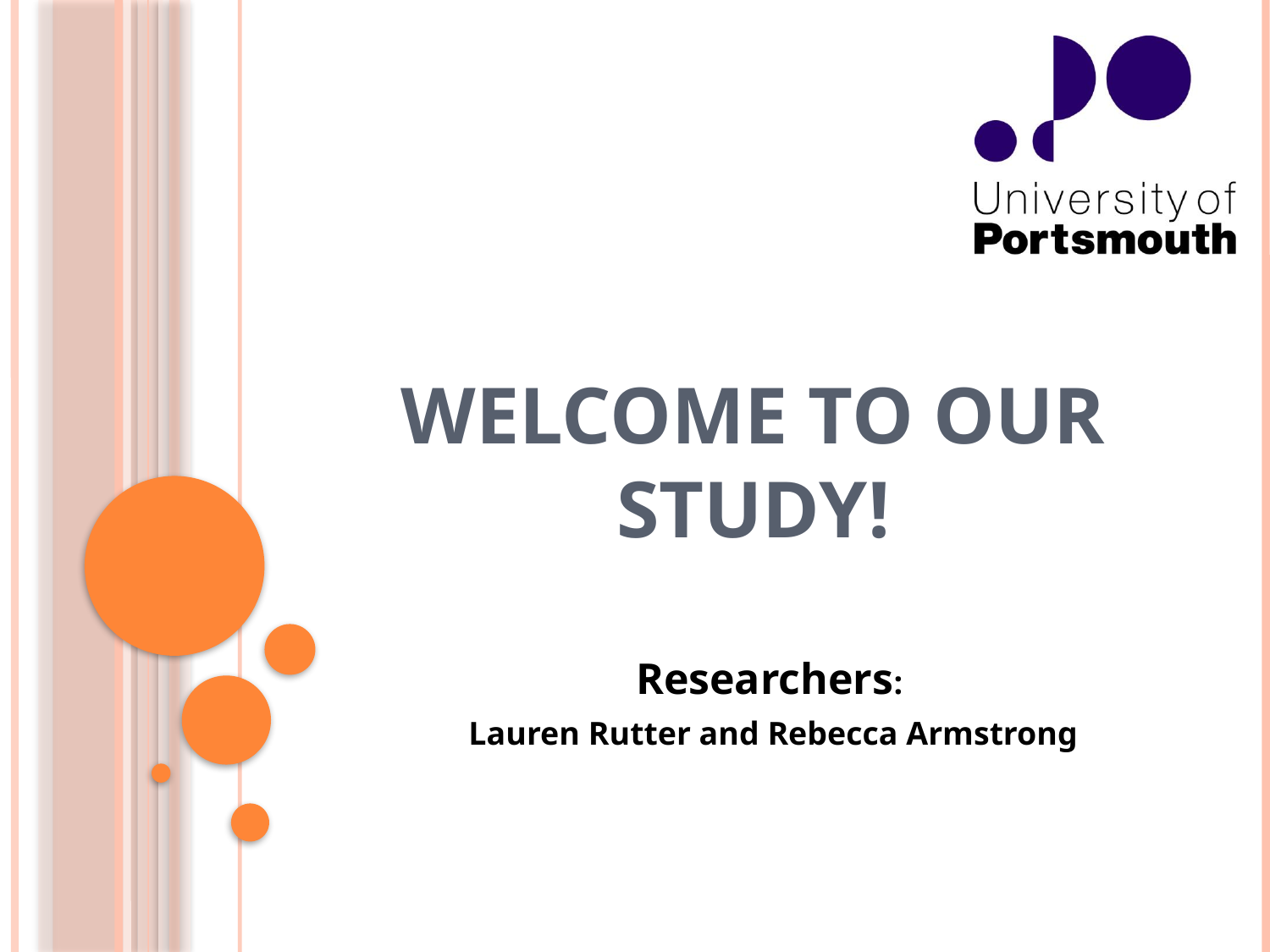

# Welcome to our study!
Researchers:
Lauren Rutter and Rebecca Armstrong

## Slide 2
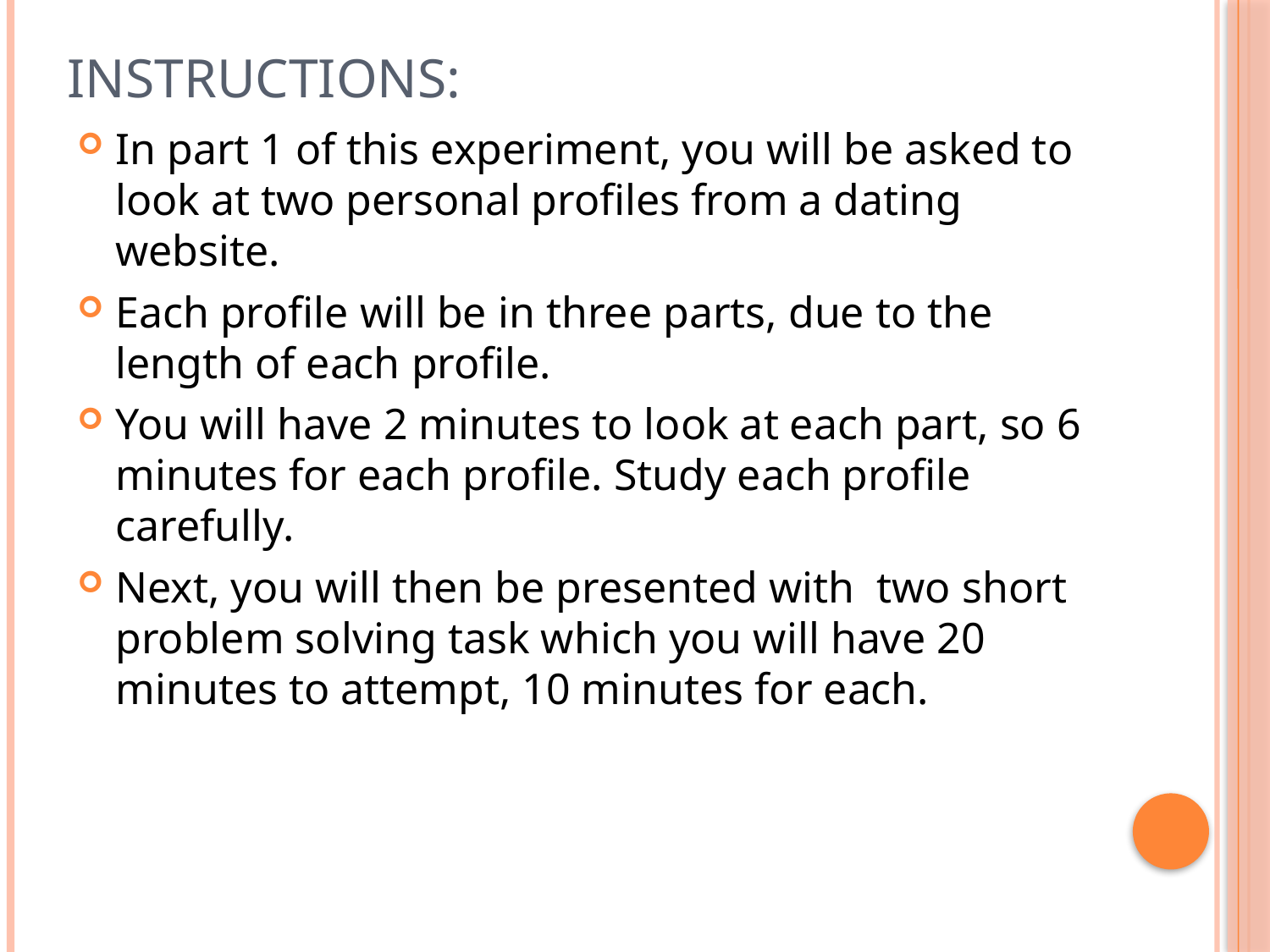

# Instructions:
In part 1 of this experiment, you will be asked to look at two personal profiles from a dating website.
Each profile will be in three parts, due to the length of each profile.
You will have 2 minutes to look at each part, so 6 minutes for each profile. Study each profile carefully.
Next, you will then be presented with two short problem solving task which you will have 20 minutes to attempt, 10 minutes for each.

## Slide 3
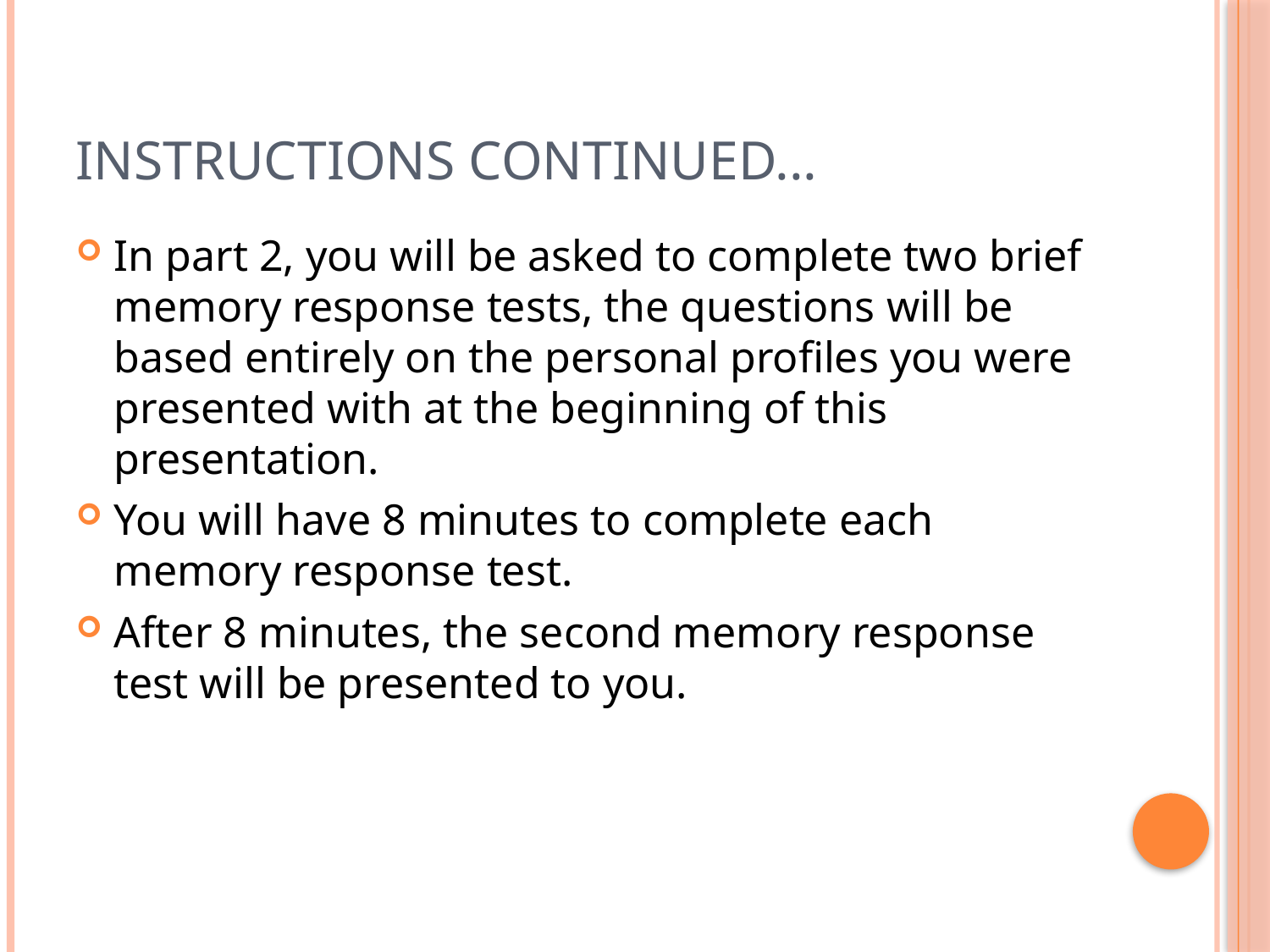

# Instructions continued...
In part 2, you will be asked to complete two brief memory response tests, the questions will be based entirely on the personal profiles you were presented with at the beginning of this presentation.
You will have 8 minutes to complete each memory response test.
After 8 minutes, the second memory response test will be presented to you.

## Slide 4
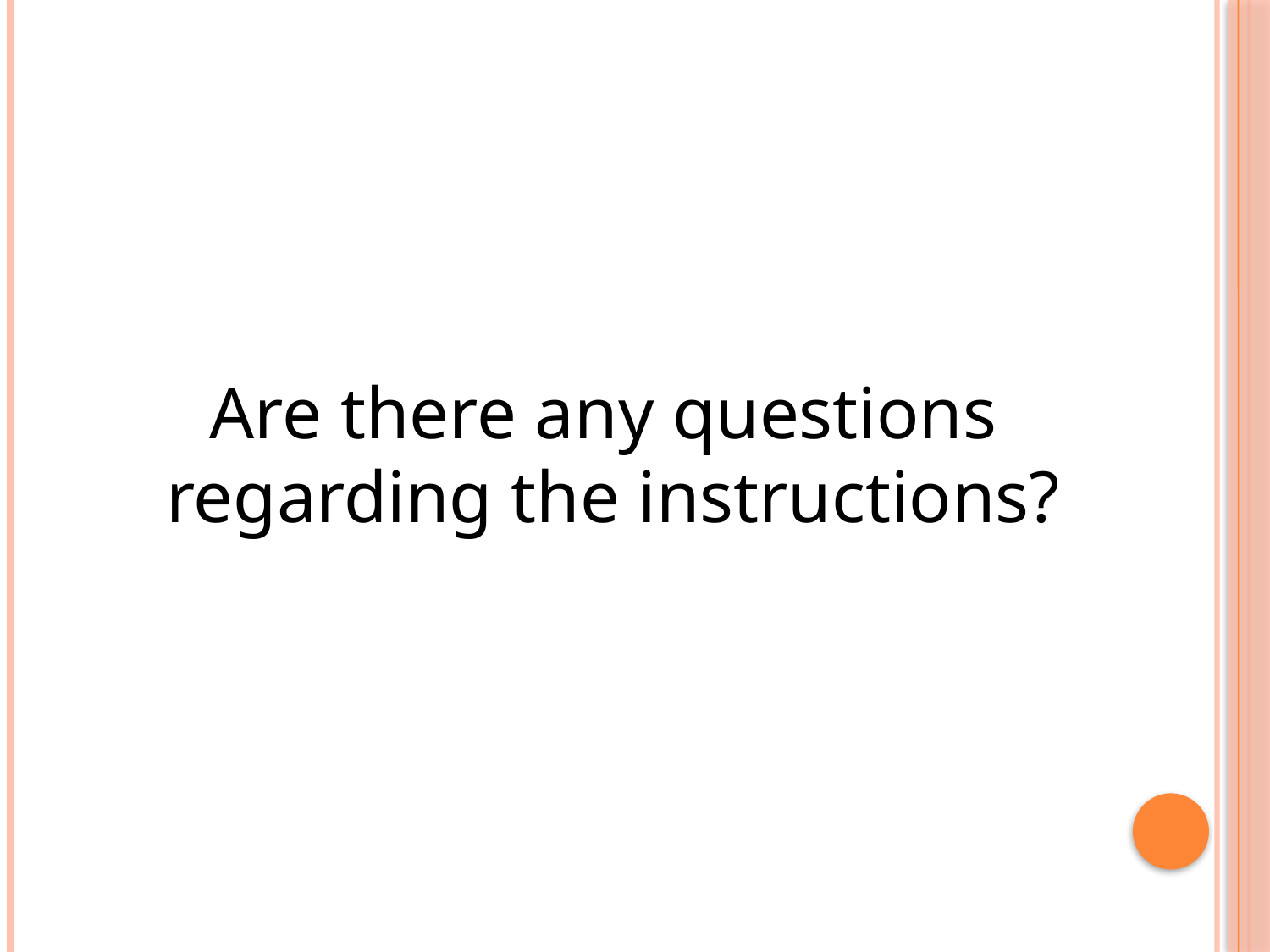

Are there any questions regarding the instructions?

## Slide 5
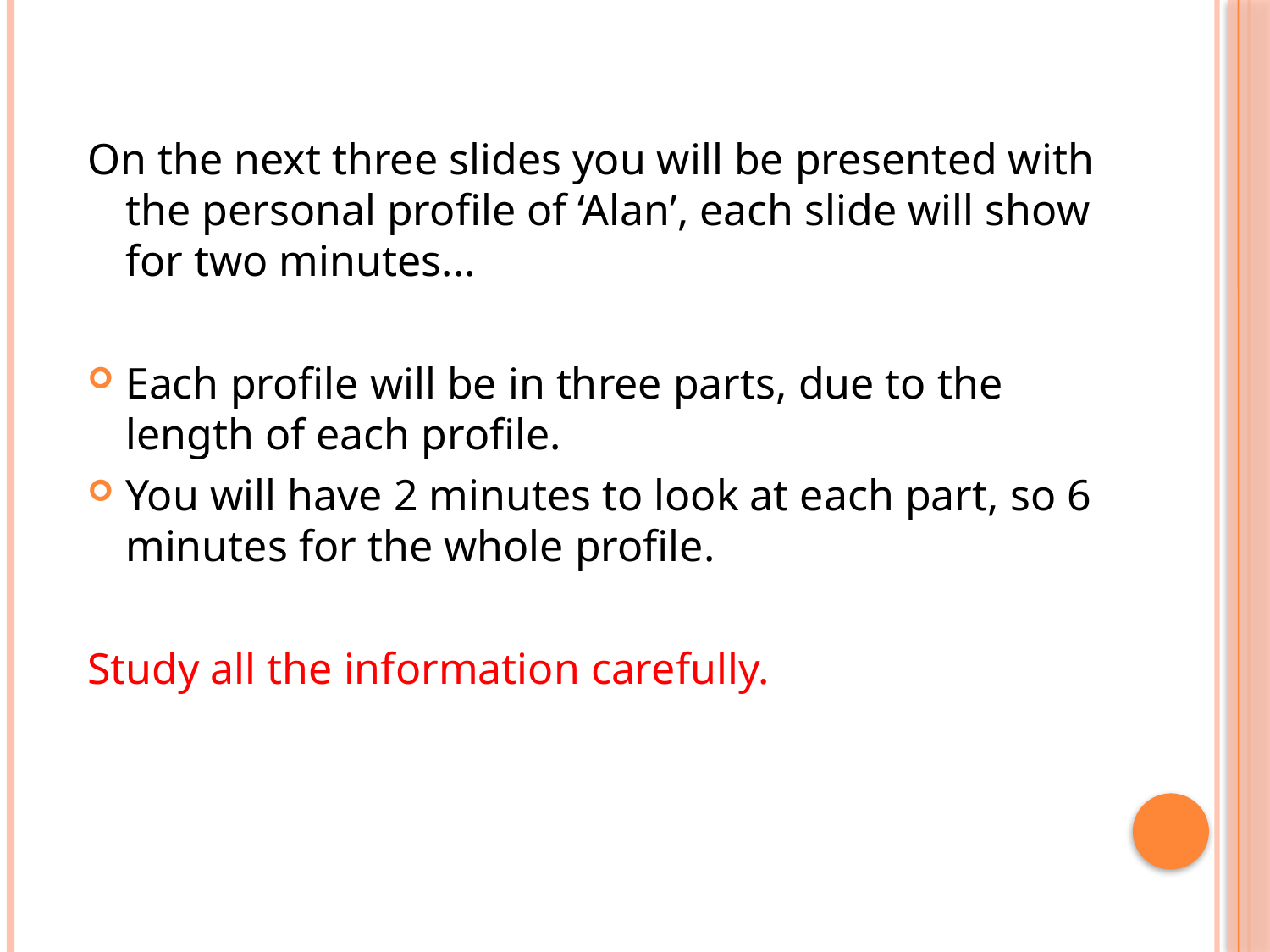

On the next three slides you will be presented with the personal profile of ‘Alan’, each slide will show for two minutes...
Each profile will be in three parts, due to the length of each profile.
You will have 2 minutes to look at each part, so 6 minutes for the whole profile.
Study all the information carefully.

## Slide 6
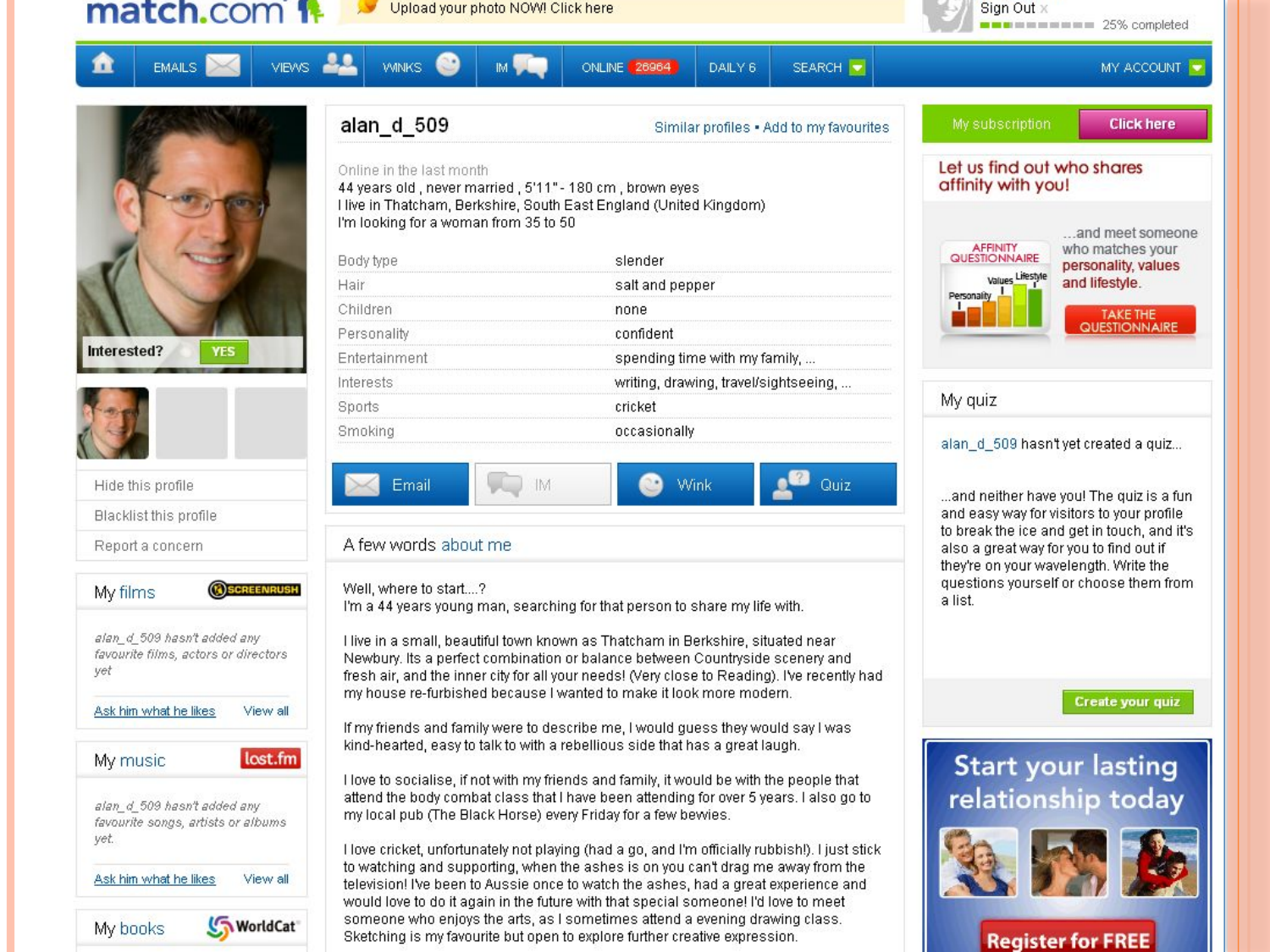

## Slide 7
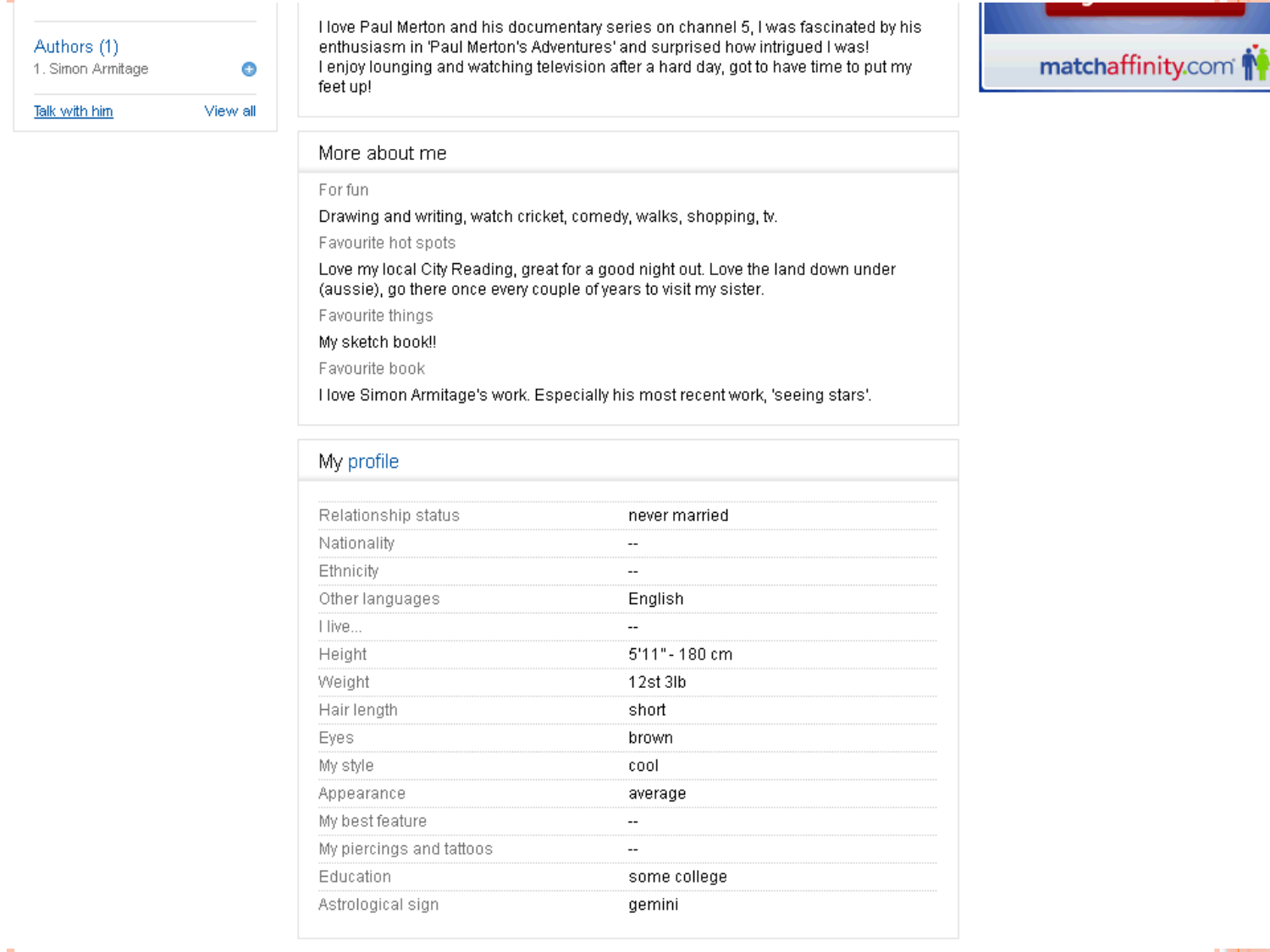

## Slide 8
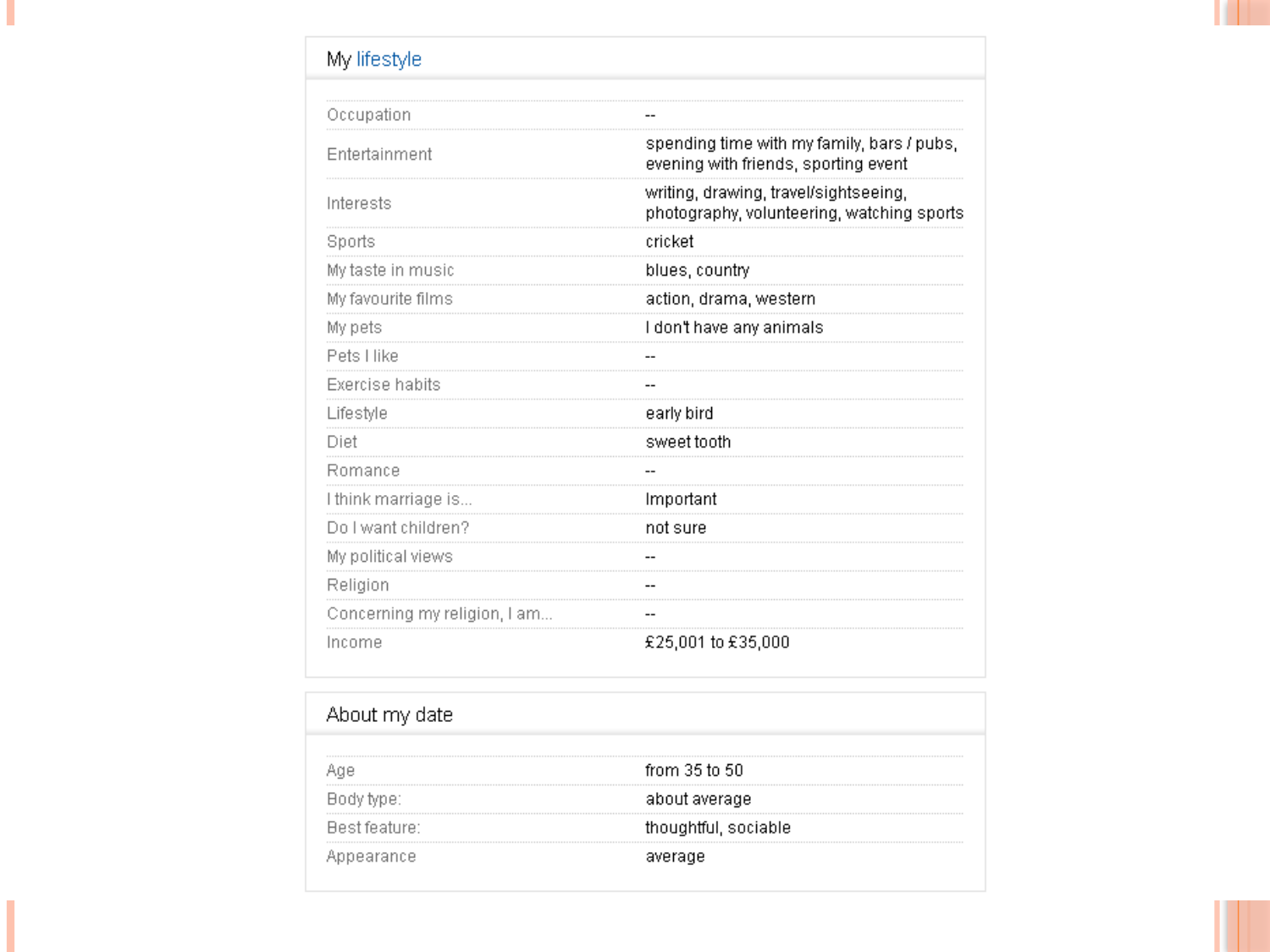

## Slide 9
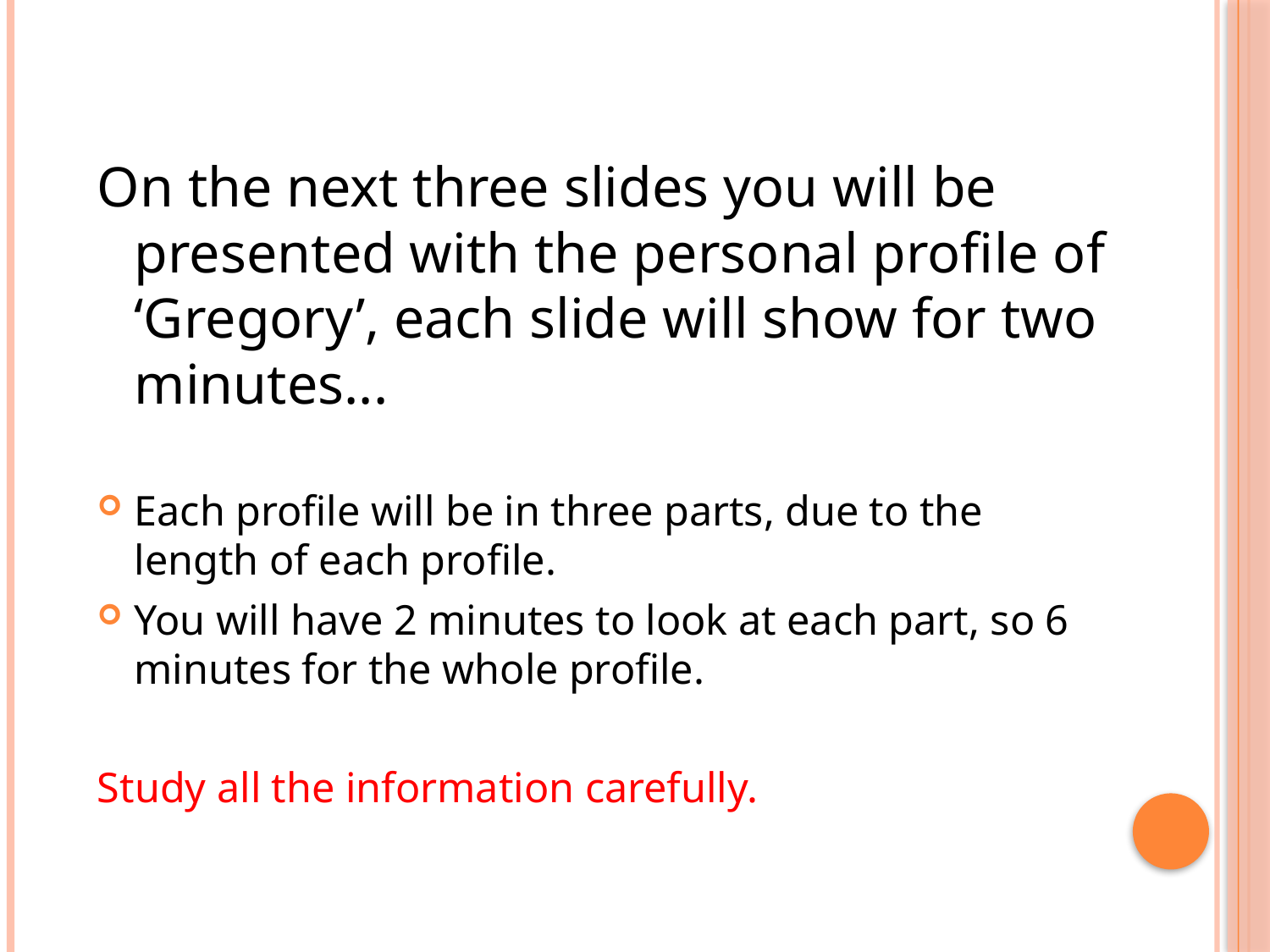

On the next three slides you will be presented with the personal profile of ‘Gregory’, each slide will show for two minutes...
Each profile will be in three parts, due to the length of each profile.
You will have 2 minutes to look at each part, so 6 minutes for the whole profile.
Study all the information carefully.

## Slide 10
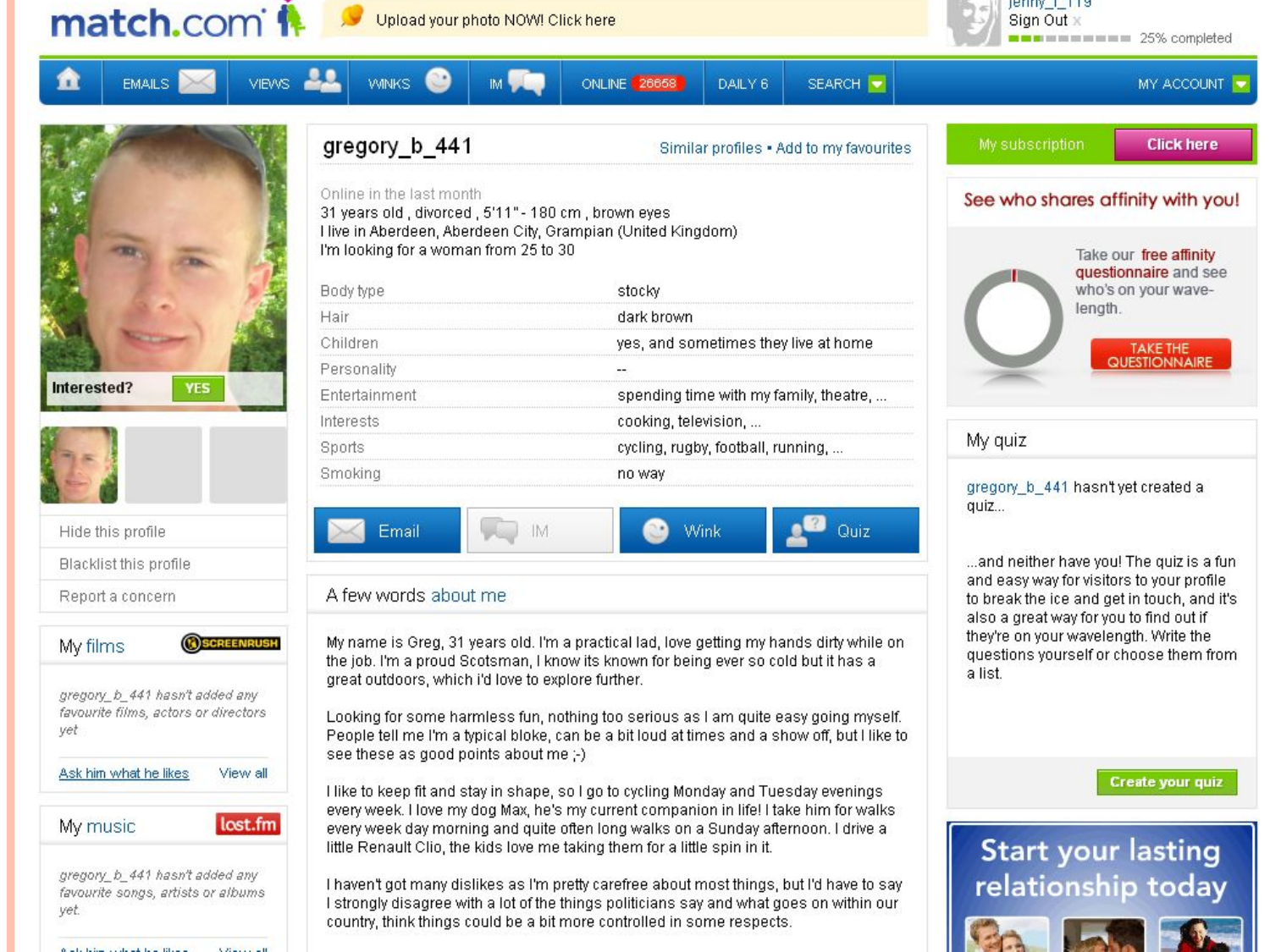

## Slide 11
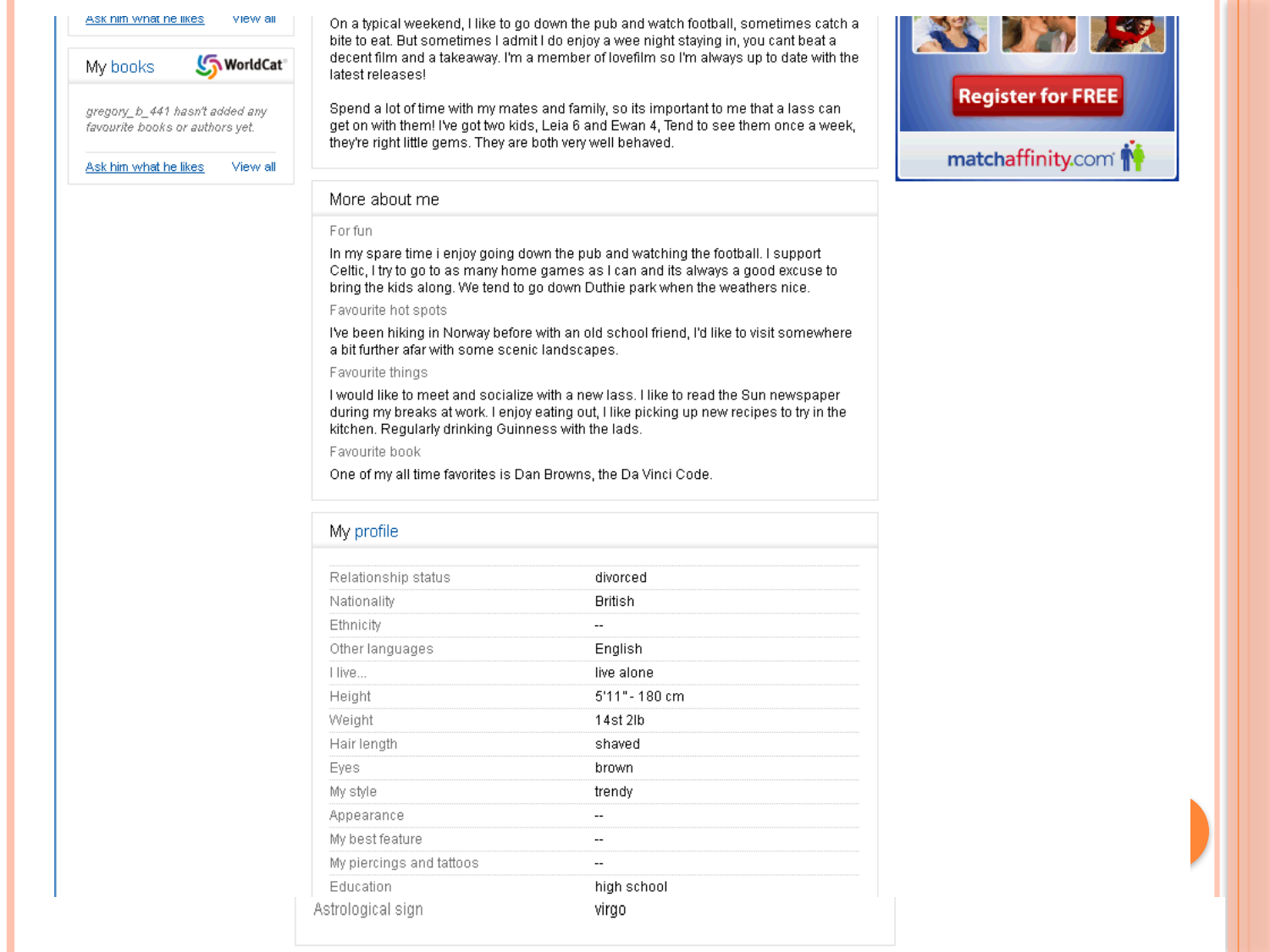

## Slide 12
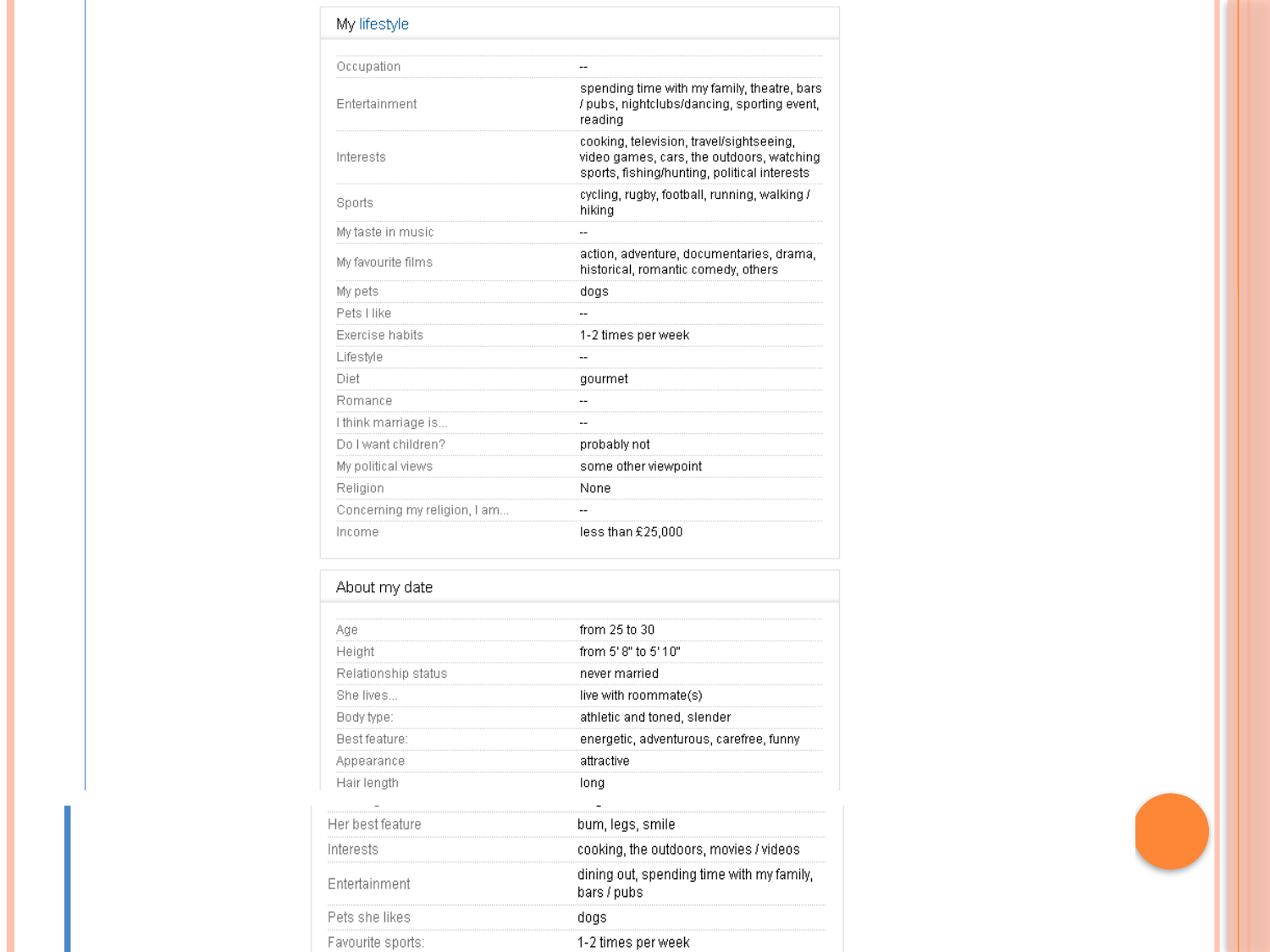

## Slide 13
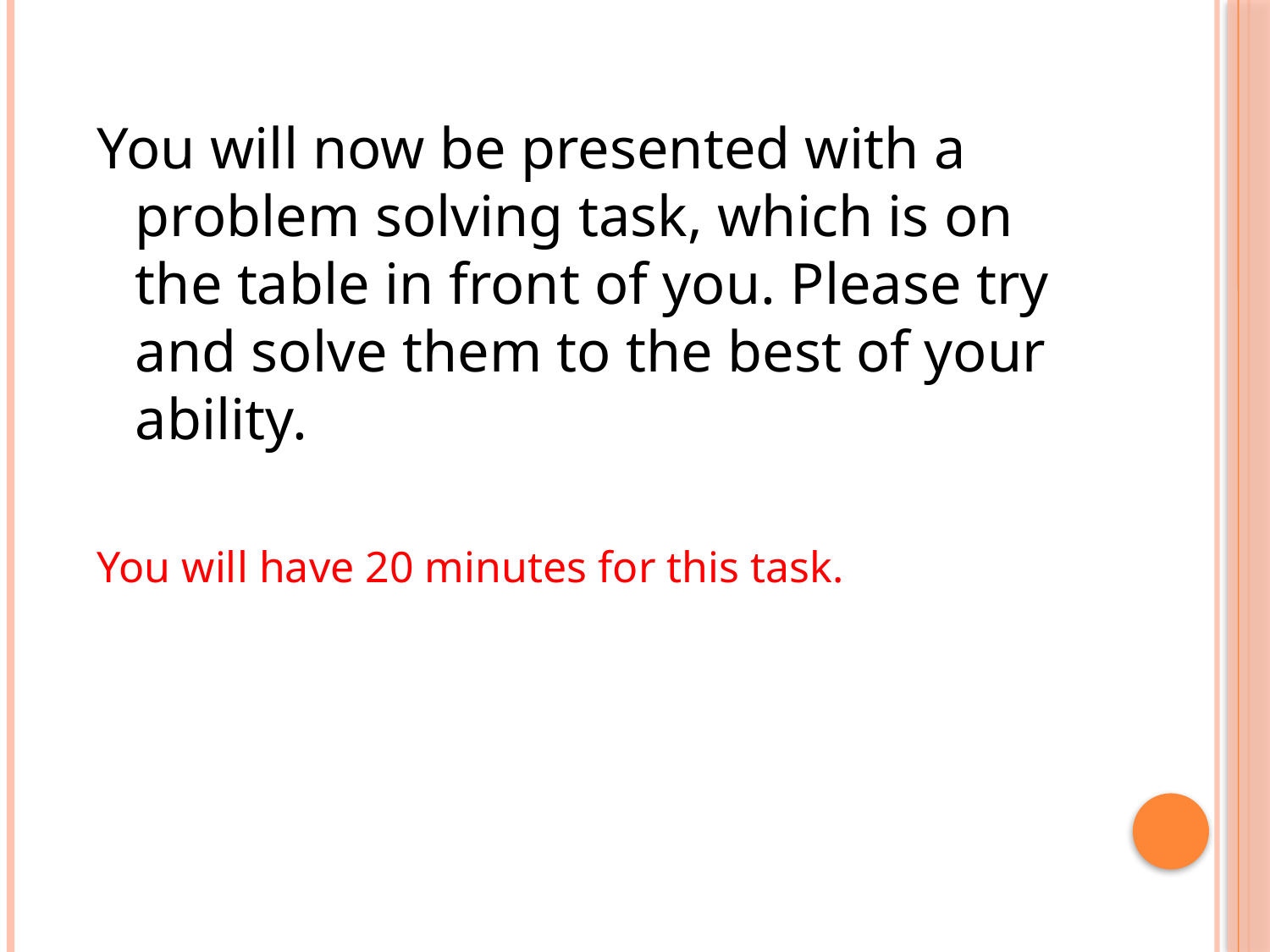

You will now be presented with a problem solving task, which is on the table in front of you. Please try and solve them to the best of your ability.
You will have 20 minutes for this task.

## Slide 14
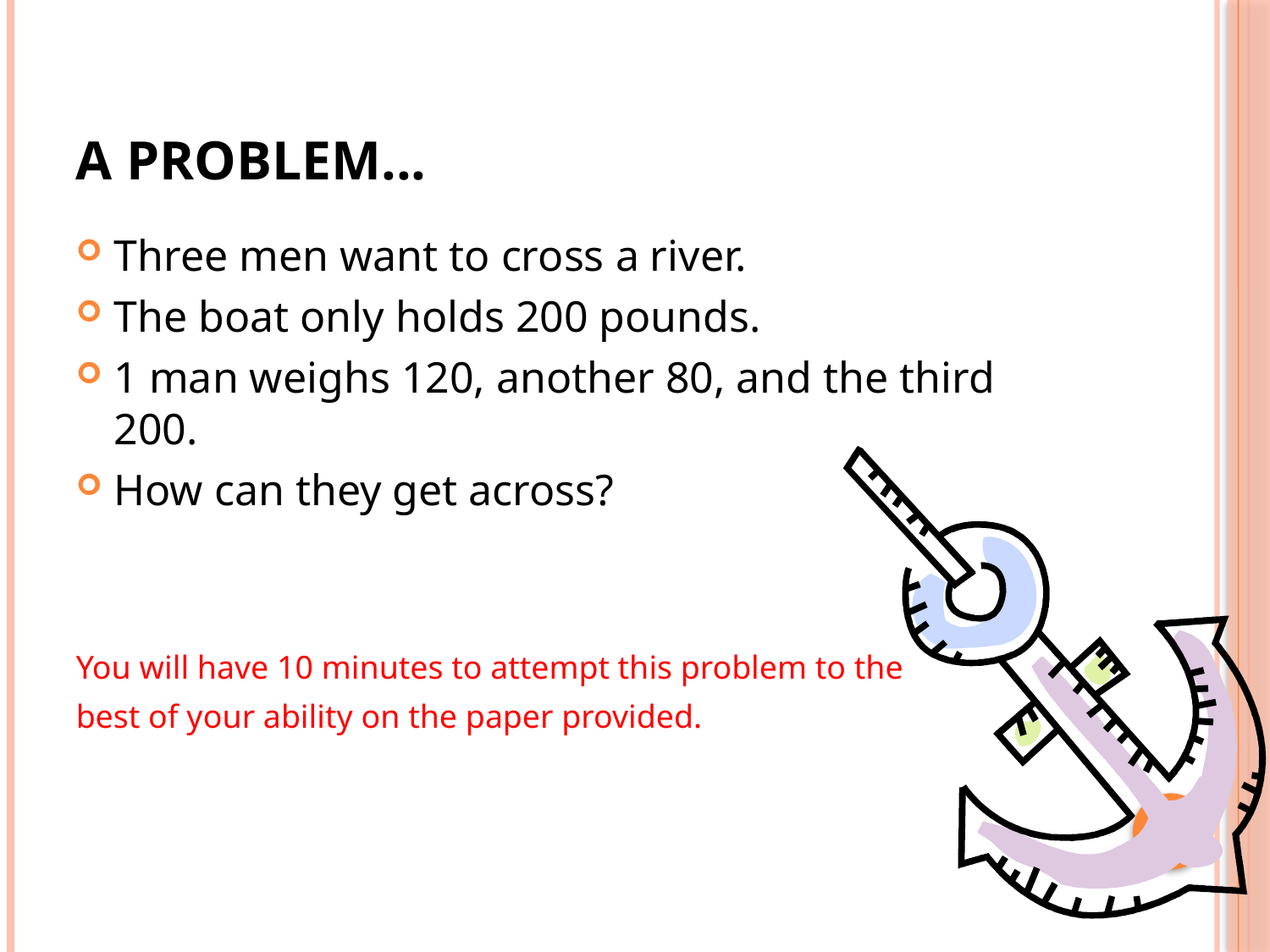

# A problem...
Three men want to cross a river.
The boat only holds 200 pounds.
1 man weighs 120, another 80, and the third 200.
How can they get across?
You will have 10 minutes to attempt this problem to the
best of your ability on the paper provided.

## Slide 15
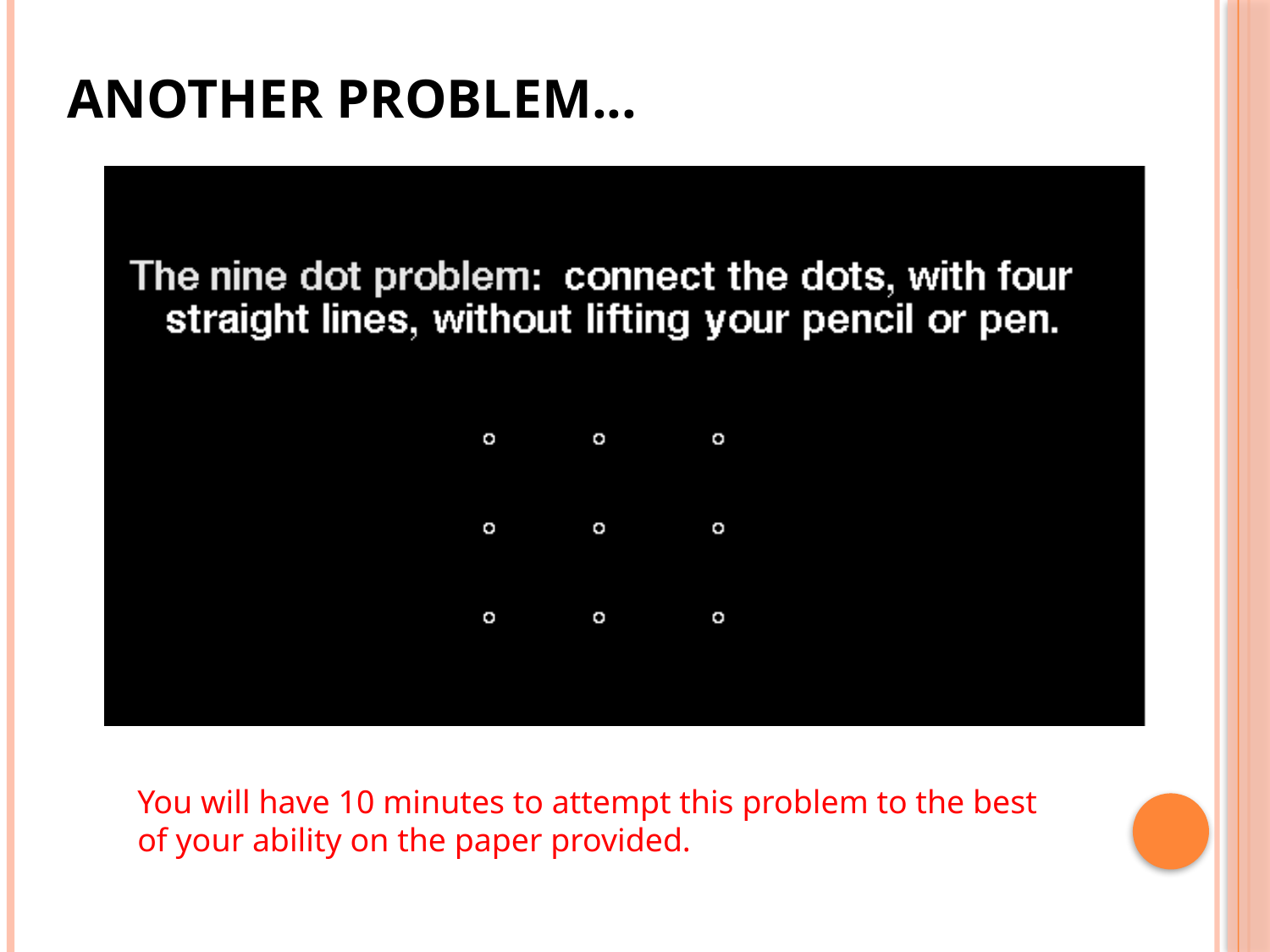

# Another problem...
You will have 10 minutes to attempt this problem to the best of your ability on the paper provided.

## Slide 16
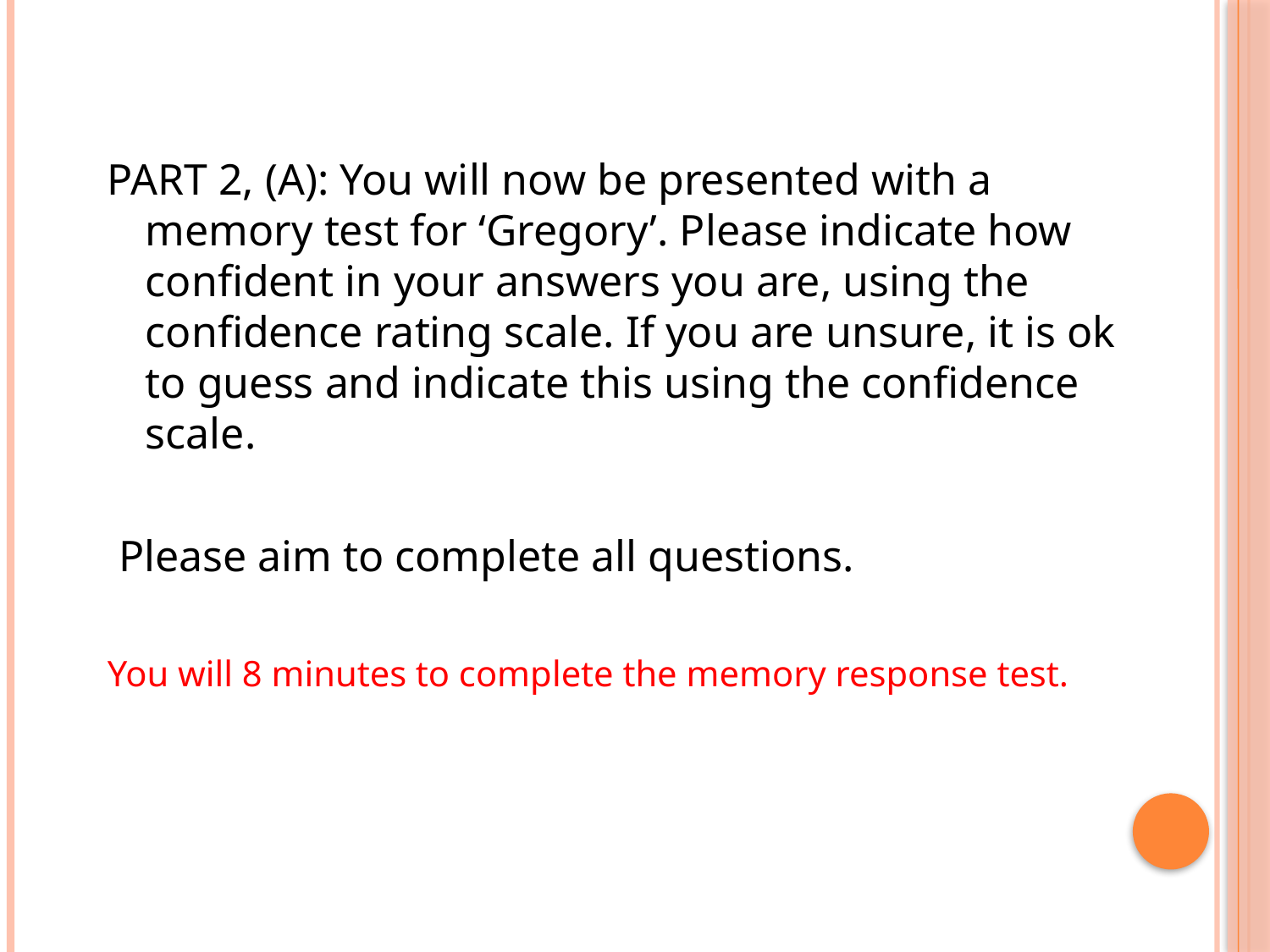

PART 2, (A): You will now be presented with a memory test for ‘Gregory’. Please indicate how confident in your answers you are, using the confidence rating scale. If you are unsure, it is ok to guess and indicate this using the confidence scale.
 Please aim to complete all questions.
You will 8 minutes to complete the memory response test.

## Slide 17
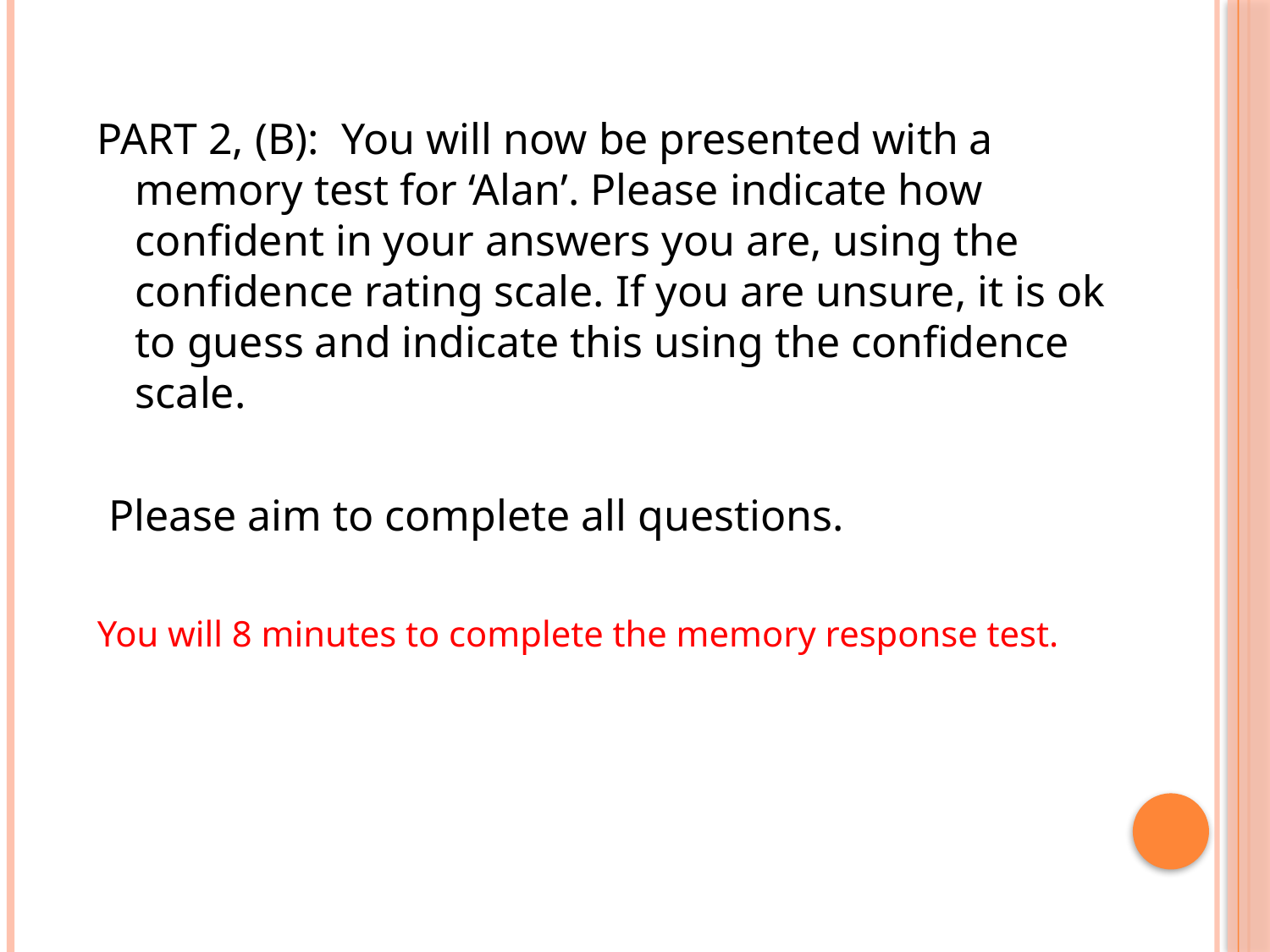

PART 2, (B): You will now be presented with a memory test for ‘Alan’. Please indicate how confident in your answers you are, using the confidence rating scale. If you are unsure, it is ok to guess and indicate this using the confidence scale.
 Please aim to complete all questions.
You will 8 minutes to complete the memory response test.

## Slide 18
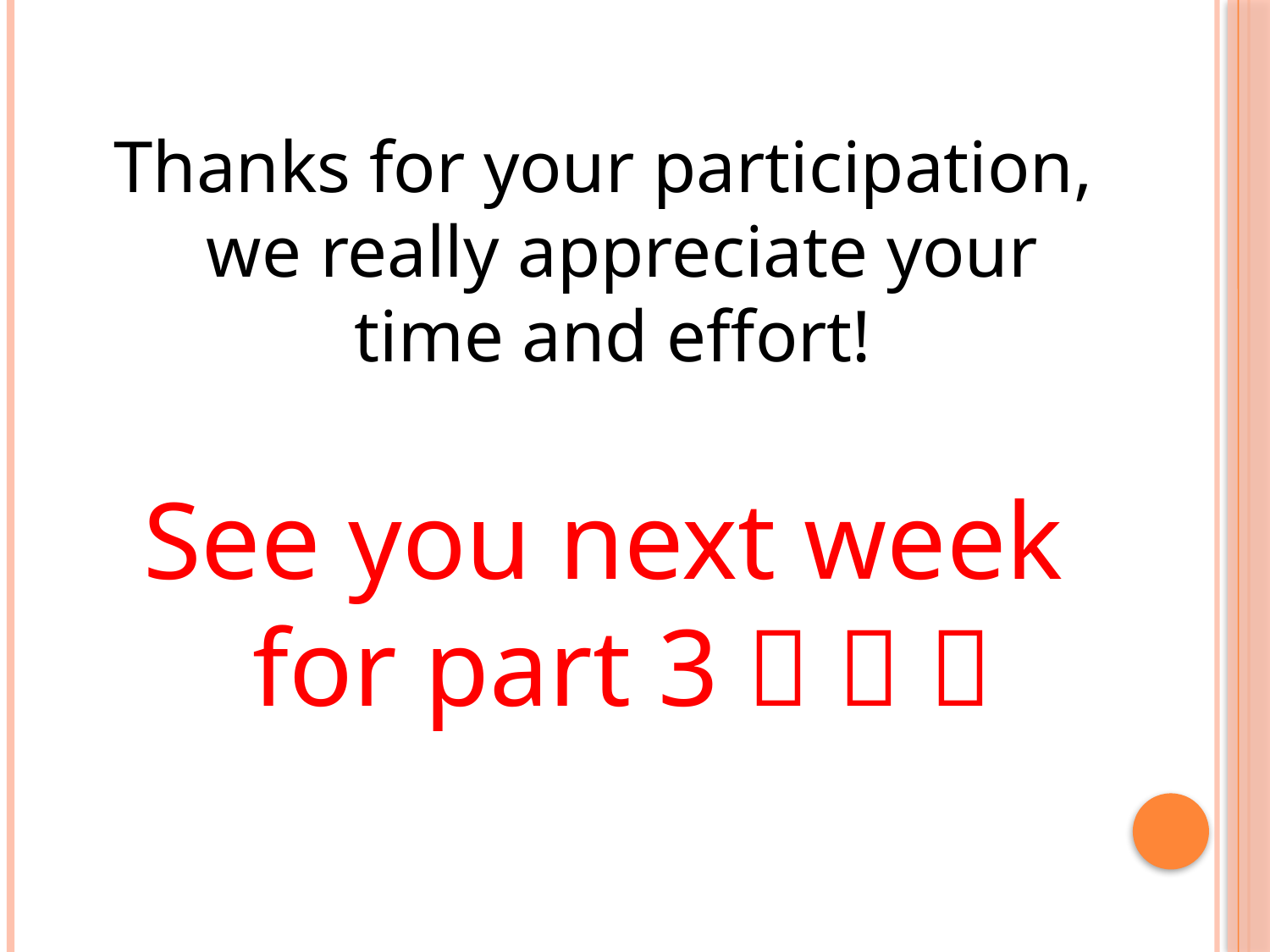

Thanks for your participation, we really appreciate your time and effort!
See you next week for part 3   
