# Supplementary material for: Enlightenment beats prejudice: The reversibility of stereotype-induced memory distortion
Source: Psychon Bull Rev. 2019 Jan 2;26(3):1001–7. doi: 10.3758/s13423-018-1541-7 (PMC6557864; doi:10.3758/s13423-018-1541-7)
Supplement: Supplementary file 1 — (ZIP 2.02 mb) [file 13423_2018_1541_MOESM1_ESM.zip › supplementary 11-27-18/Memory test Alan (occupation label version).pdf]

P1

Participant No: 1

## Memory response test

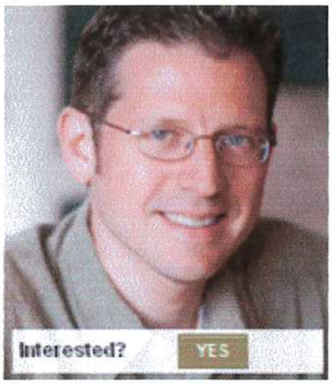


**Username:** Alan_d_509

**Gender:** Male

**Occupation:** Vicar

Age: 20

Gender: M

The following questions are based on information you have received about Alan, see above for a reminder of who Alan is.

**In this questionnaire, you will be presented with twenty questions regarding various things about Alan. Alan is shown in the picture above, you also read over a personal profile on him earlier. Please now answer each of the following questions carefully, as correctly as you can based on all the information that has been presented to you about Alan.**

**Please circle your chosen answers.**

**1. Where does Alan live?**

- Southampton, Hampshire
- Cardiff (Wales)
- (circled) Thatcham, Berkshire
- Dublin (Ireland)

Not at all confident — Very confident

|____|____|____|____|

1 2 3 4 (5, circled)

**2. Where does Alan go to visit his sister?**

- Scotland
- North London
- Australia
- France

Not at all confident | Very confident

|_______|_______|_______|_______|

1 2 3 4 5

**3. What does Alan do every Friday night?**

- Watch game shows
- Go clubbing
- Go to his local pub
- Community work

Not at all confident | Very confident

|_______|_______|_______|_______|

1 2 3 4 5

**4. What does Alan describe his house as?**

- A council flat
- Recently re-furbished and modern
- A house in the country
- A traditional house with old furnishings

Not at all confident | Very confident

|_______|_______|_______|_______|

1 2 3 4 5

**5. What is Alan's local pub called?**

- The Good Intent
- The Ship and Bell
- The Black Horse
- The Dog and Bacon

Not at all confident Very confident

|____|____|____|____|

1 2 3 4 5

**6. What class has Alan been attending for over five years?**

- Judo
- Squash
- Body Combat
- Swimming

Not at all confident Very confident

|____|____|____|____|

1 2 3 4 5

**7. What is Alan's favourite sport?**

- Cricket
- Football
- Rugby
- Motor Racing

Not at all confident Very confident

|____|____|____|____|

1 2 3 4 5

**8. What does Alan as his view on marriage?**

- Important
- Doesn't have an opinion on marriage
- Not necessary
- Sacred

Participant No: ___

Not at all confident | | | | Very confident

|_________|_________|_________|_________|

1 2 3 (4) 5

**9. What genre of music is Alan interested in?**

- Classical music
- (Blues and Country)
- Pop and RnB
- Old school rock

Not at all confident | | | | Very confident

|_________|_________|_________|_________|

1 2 3 4 (5)

**10. What is Alan's 'astrological star sign'?**

- Scorpio
- Gemini
- (Aquarius)
- Cancer

Not at all confident | | | | Very confident

|_________|_________|_________|_________|

1 2 3 (4) 5

**11. What is Alan's highest level of qualification?**

- B-tech in social studies
- (Some college qualifications)
- Masters or PHD
- Honours Degree

Not at all confident | | | | Very confident

|_________|_________|_________|_________|

1 2 3 4 (5)

Participant No: ___

**12. Does Alan smoke?**

- Never
- Occasionally
- Yes
- Attempting to quit

Not at all confident Very confident

|________|________|________|________|

1 2 3 4 5

**13. What documentary did Alan say he enjoys watching?**

- 'Frozen Planet' with David Attenborough
- 'An Idiot Abroad' with Karl Pilkington
- 'Wonders of the Universe' with Brian Cox
- Paul Merton's adventures

Not at all confident Very confident

|________|________|________|________|

1 2 3 4 5

**14. What is Alan's 'favourite thing'?**

- His computer
- Bible
- Football boots
- Sketch book

Not at all confident Very confident

|________|________|________|________|

1 2 3 4 5

**15. What does Alan state as his typical 'diet'?**

- Reasonably healthy
- Simplistic
- Sweet tooth

- Lots of takeaway food

Not at all confident Very confident

| 1 | 2 | 3 | 4 | 5 |
|---|---|---|---|---|

**16. What does Alan state as his 'personality type'?**

- Shy
- Confident
- Stubborn
- Helpful / advising

Not at all confident Very confident

| 1 | 2 | 3 | 4 | 5 |
|---|---|---|---|---|

**17. What does Alan describe as his 'typical dress style'?**

- Cool
- Jeans and t-shirt
- Black suits
- Sportswear

Not at all confident Very confident

| 1 | 2 | 3 | 4 | 5 |
|---|---|---|---|---|

**18. What is Alan's favourite book?**

- 'No time for goodbye' by Linwood Barclay
- Doesn't read books
- 'Water for elephants' by Sara Gruen
- 'Seeing stars' by Simon Armitage

Not at all confident Very confident

|_____|_____|_____|_____|

1 2 3 4 5

**19. How old did Alan say he was?**

- 44
- 32
- 28
- 50 +

Not at all confident Very confident

|_____|_____|_____|_____|

1 2 3 4 5

**20. What is Alan's relationship status?**

- married
- Currently separated
- Never been married
- Divorced

Not at all confident Very confident

|_____|_____|_____|_____|

1 2 3 4 5
