# Supplementary material for: Enlightenment beats prejudice: The reversibility of stereotype-induced memory distortion
Source: Psychon Bull Rev. 2019 Jan 2;26(3):1001–7. doi: 10.3758/s13423-018-1541-7 (PMC6557864; doi:10.3758/s13423-018-1541-7)
Supplement: Supplementary file 1 — (ZIP 2.02 mb) [file 13423_2018_1541_MOESM1_ESM.zip › supplementary 11-27-18/Memory test Greg (no label version).pdf]

P1

Participant No: (1)

Memory response test

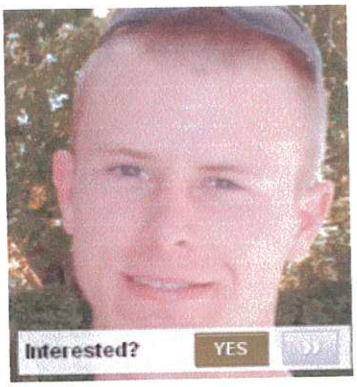

Username: Gregory_b_441

Gender: Male

Age: 20

Gender: M

The following questions are based on information you have received about Gregory, see above for a reminder of who Gregory is.

In this questionnaire, you will be presented with twenty questions regarding various things about Greg. Greg is shown in the picture above, you also read over a personal profile on him earlier. Please now answer each of the following questions carefully, as accurately as you can based on all the information that has been presented to you about Greg.

Please circle your chosen answers.

1. Does Greg smoke?

- Yes
- Occasionally
- No (circled)
- Attempting to quit

Not at all confident — Very confident

|_____|_____|_____|_____|

1 2 3 4 5 (5 circled)

2. **What newspaper does Greg like to read during his breaks at work?**

- Daily star
- The sun
- Guardian
- Local newspaper

Not at all confident Very confident

|_____|_____|_____|_____|

1 2 3 4 5

3. **Where does Greg live?**

- Harlow, Essex (North London)
- Aberdeen, Grampian
- Margate (Kent)
- Bristol City

Not at all confident Very confident

|_____|_____|_____|_____|

1 2 3 4 5

4. **What vehicle does Greg say he drives?**

- Land rover
- Renault Clio
- White van
- Nissan Micra

Not at all confident Very confident

|_____|_____|_____|_____|

1 2 3 4 5

5. **How old is Greg ?**

- 28
- 36
- 33
- 31

Not at all confident | Very confident

|________|________|________|________|

1 2 3 4 5

6. **What does Greg like to do on a weekend?**

- Go to a music gig
- Playing games consoles on xbox
- Watching a film
- Exercise class

Not at all confident | Very confident

|________|________|________|________|

1 2 3 4 5

7. **What does Greg do on a Monday and Tuesday evening every week?**

- Go to the pub quiz
- Work on his car
- Go cycling
- Watch the tv

Not at all confident | Very confident

|________|________|________|________|

1 2 3 4 5

Participant No: ___

8. What alcoholic drink does Greg drink when he goes down the pub to watch football?

- Wine
- Lager / beer
- Guinness
- Cider

Not at all confident   Very confident

|________|________|________|________|

1   2   3   4   5

9. What football team does Greg support?

- Celtic
- Stoke
- Manchester United
- Tottenham Hotspur

Not at all confident   Very confident

|________|________|________|________|

1   2   3   4   5

10. What would Greg do if he visited Norway?

- Skiing
- Hiking
- To see the Fjord's
- Go to the Viking museum / exhibitions

Not at all confident   Very confident

|________|________|________|________|

1   2   3   4   5

11. What does Greg state that he dislikes most?

Participant No: ___

- Politicians
- Overweight people
- Homosexuals
- Snobby people

Not at all confident | Very confident

|________|________|________|________|

1 2 3 4 5

12. What is Greg's current relationship status?

- Widowed
- Divorced
- Currently separated
- Never been married

Not at all confident | Very confident

|________|________|________|________|

1 2 3 4 5

13. What traits does Greg admit to having?

- Loud and show off
- Lazy
- Caring
- Funny

Not at all confident | Very confident

|________|________|________|________|

1 2 3 4 5

14. What are Greg's two children called?

- Jamie and Isabella
- Chris and Sarah
- Leia and Ewan
- Hayley and George

Not at all confident — Very confident

|_____|_____|_____|_____|

1 2 3 4 5

15. What is Greg's favourite book?

- Doesn't read books
- Great expectations – Charles Dickinson
- Trainspotting - Irvine Welsh
- Da Vinci Code – Dan Brown

Not at all confident — Very confident

|_____|_____|_____|_____|

1 2 3 4 5

16. What is Greg's highest level of qualification?

- A levels
- Degree
- Diploma
- High school

Not at all confident — Very confident

|_____|_____|_____|_____|

1 2 3 4 5

Participant No: ___

17. What does Greg look for as a 'Best feature' in his potential new partner?

- Legs
- Breasts
- Lips
- Eyes

Not at all confident — Very confident

|____|____|____|____|

1 2 3 4 5

18. What does Greg typically eat in his diet?

- Fast food
- Meat and potatoes
- Gourmet food
- A careful diet

Not at all confident — Very confident

|____|____|____|____|

1 2 3 4 5

19. What does Greg state as his 'body type'?

- Stocky
- Athletic and toned
- Skinny
- Quite large, few extra pounds

Not at all confident   Very confident

|________|________|________|________|

1   2   3   (4)   5

20. What does Greg describe as his typical 'dress style'?

- (Sporty wear)
- Scruffy jeans and a t-shirt
- Rock / alternative
- Trendy

Not at all confident   Very confident

|________|________|________|________|

1   2   (3)   4   5
